# Supplementary material for: Regional occupancy increases for widespread species but decreases for narrowly distributed species in metacommunity time series
Source: Nat Commun. 2023 Mar 16;14:1463. doi: 10.1038/s41467-023-37127-2 (PMC10020147; doi:10.1038/s41467-023-37127-2)
Supplement: Supplementary file 1 — Supplementary information [file 41467_2023_37127_MOESM1_ESM.pdf]

Supplementary Information For

Regional occupancy increases for widespread species but decreases for narrowly distributed species in metacommunity time series

Wu-Bing Xu, Shane A. Blowes, Viviana Brambilla, Cher F. Y. Chow, Ada Fontrodona-Eslava, Inês S. Martins, Daniel McGlinn, Faye Moyes, Alban Sagouis, Hideyasu Shimadzu, Roel van Klink, Anne E. Magurran, Nicholas J. Gotelli, Brian J. McGill, Maria Dornelas, Jonathan M. Chase

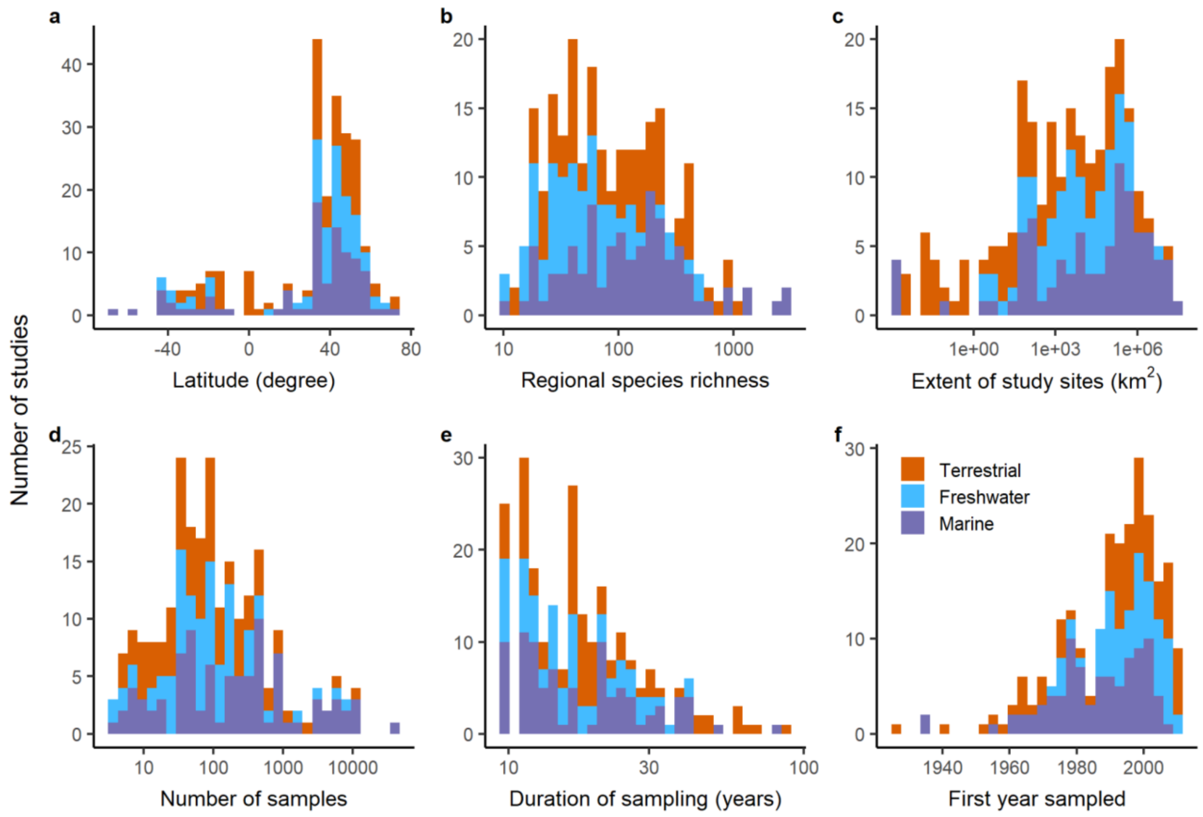

Supplementary Fig. 1 | Frequency distribution of study characteristics. a-f, The characteristics include central latitude of study sites (**a**), regional species richness observed in the study (**b**), extent of study sites (**c**), number of samples used to calculate occupancy in each period (**d**), duration of sampling (**e**), and start year of sampling (**f**). The colors indicate the terrestrial (orange), freshwater (blue) and marine (purple) realms where studies came from. The medians of these variables are 41.2° (based on absolute latitude), 76 species, 4,274 km^2, 84 samples, 16 years, and 1994, respectively.

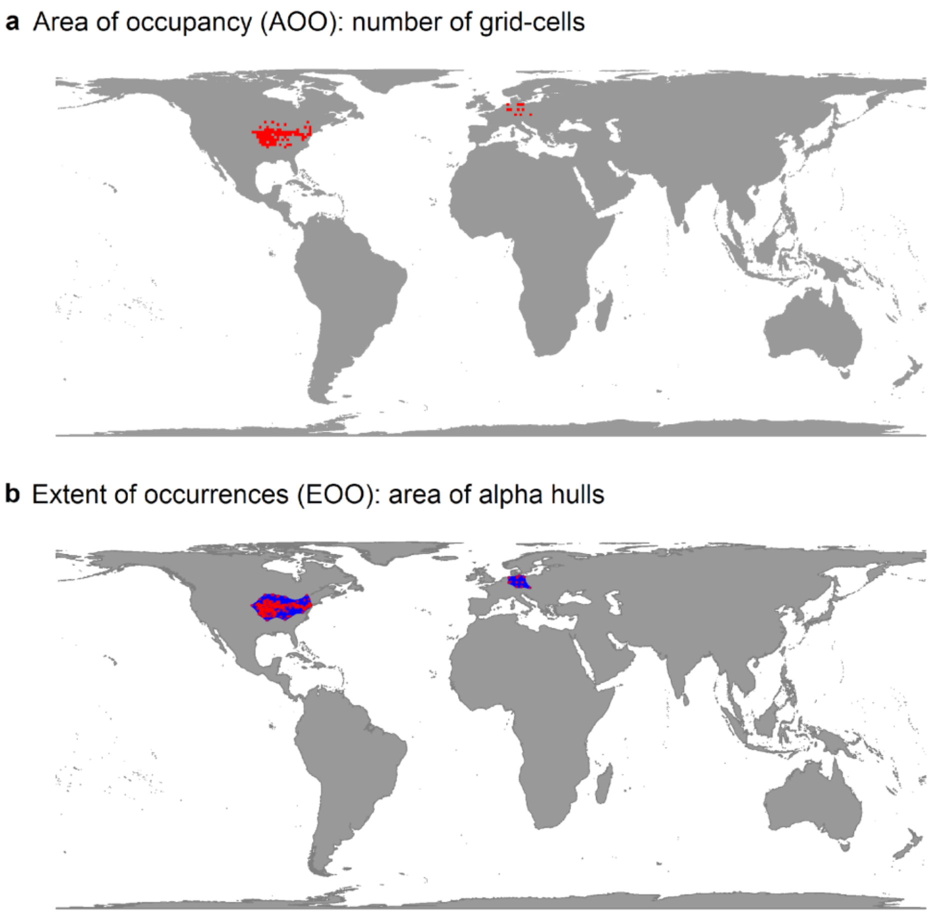

Supplementary Fig. 2 | A diagram showing how a species' geographic range size was measured. We first downloaded and cleaned species distribution occurrences from the Global Biodiversity Information Facility. A species' range size was then measured as the area of occupancy (AOO) which was estimated as the number of grid cells (red cells) occupied by the species (**a**), and the extent of occurrences (EOO) which was estimated as the area of the alpha hulls (blue polygons) containing the species' distribution occurrences (**b**). Red points in **b** were the species' distribution occurrences. We estimated AOO using three grid-cell resolutions: 10 km × 10 km, 50 km × 50 km, and 100 km × 100 km. The alpha hull was constructed using the alpha parameter set to six.

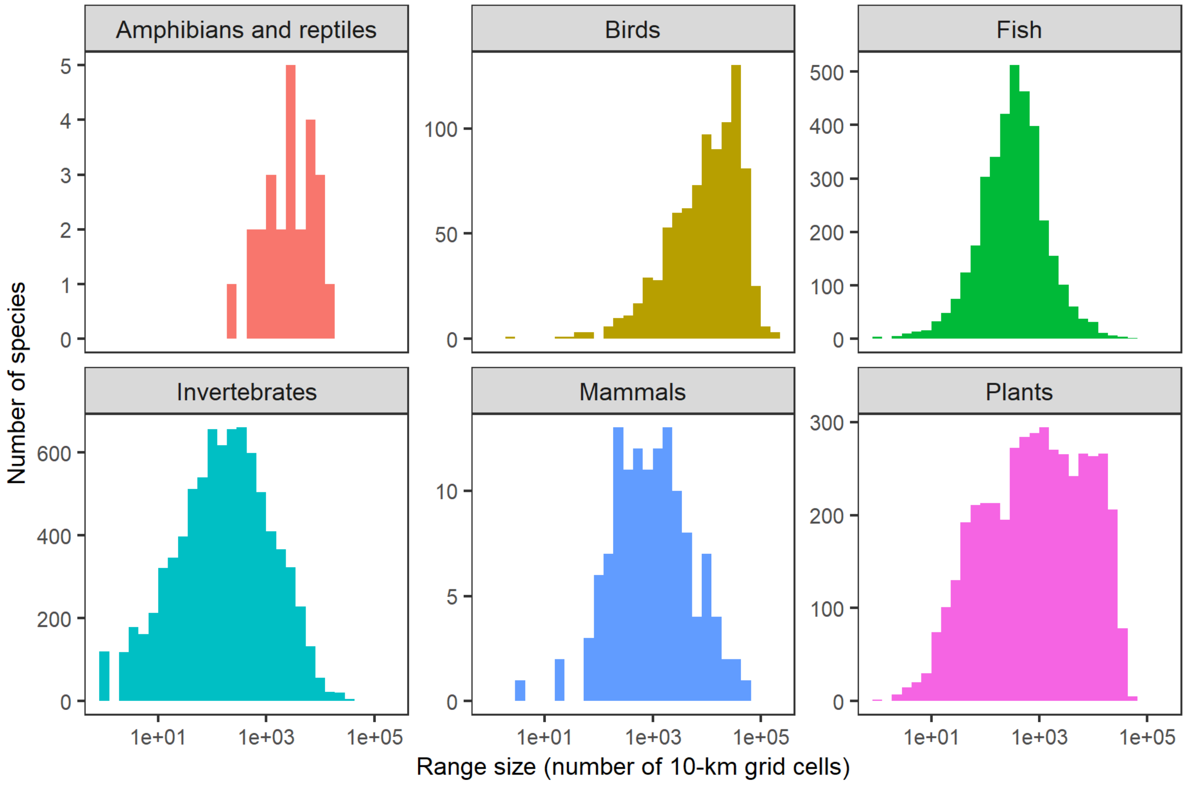

Supplementary Fig. 3 | Range size frequency distribution for each taxonomic group. Range sizes were measured as the number of 10-km grid cells where distribution occurrences were observed.

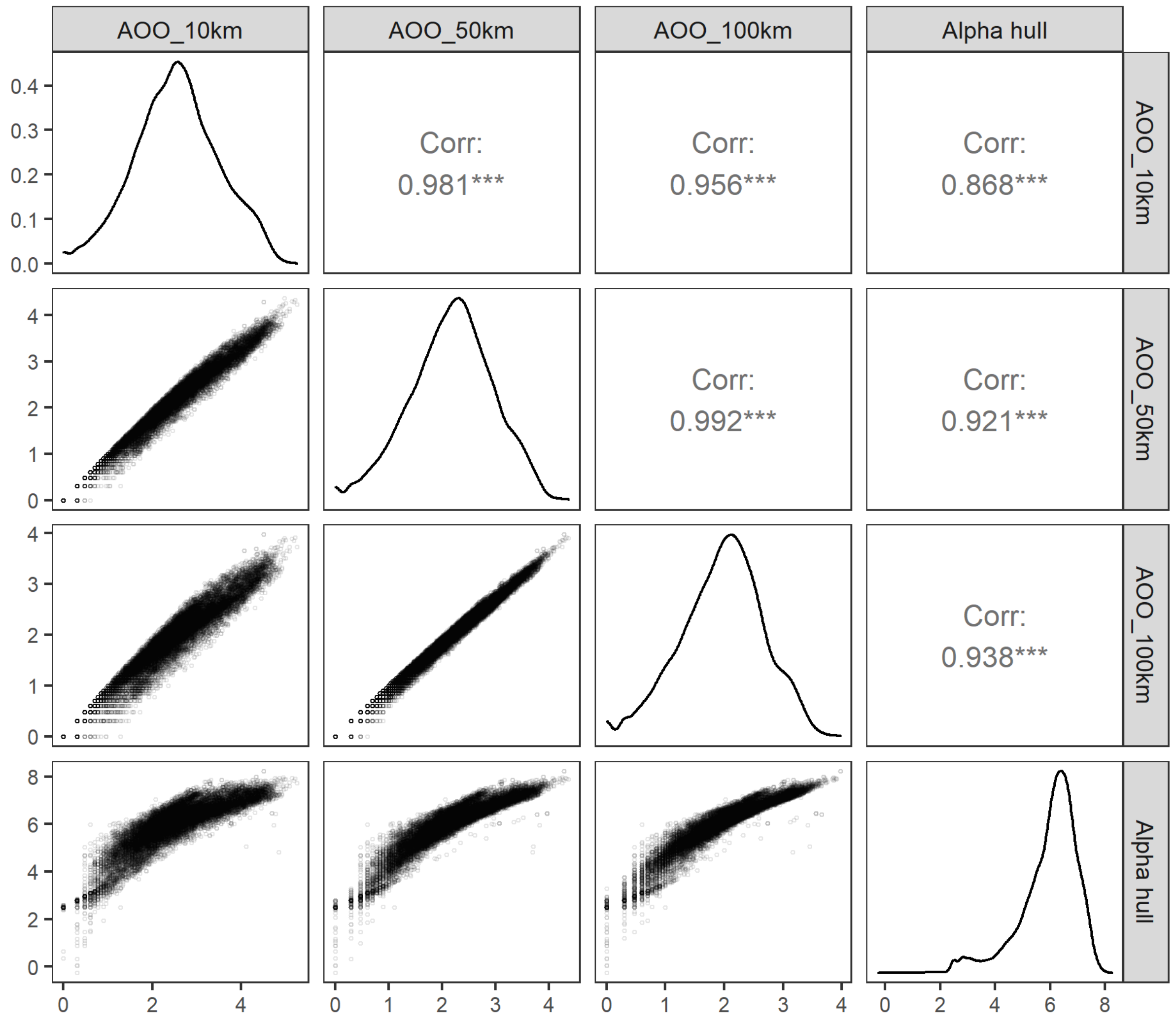

Supplementary Fig. 4 | Pearson correlation between different estimates of species' range size. Range sizes were measured as the area of occupancy (AOO) defined as the number of grid cells where distribution occurrences were observed using three resolutions (10-, 50-, 100-km), and the extent of occurrences (EOO) defined as the area of alpha hulls. There are strong positive correlations among these $\log_{10}$-transformed estimates (Pearson' $r > 0.868$).

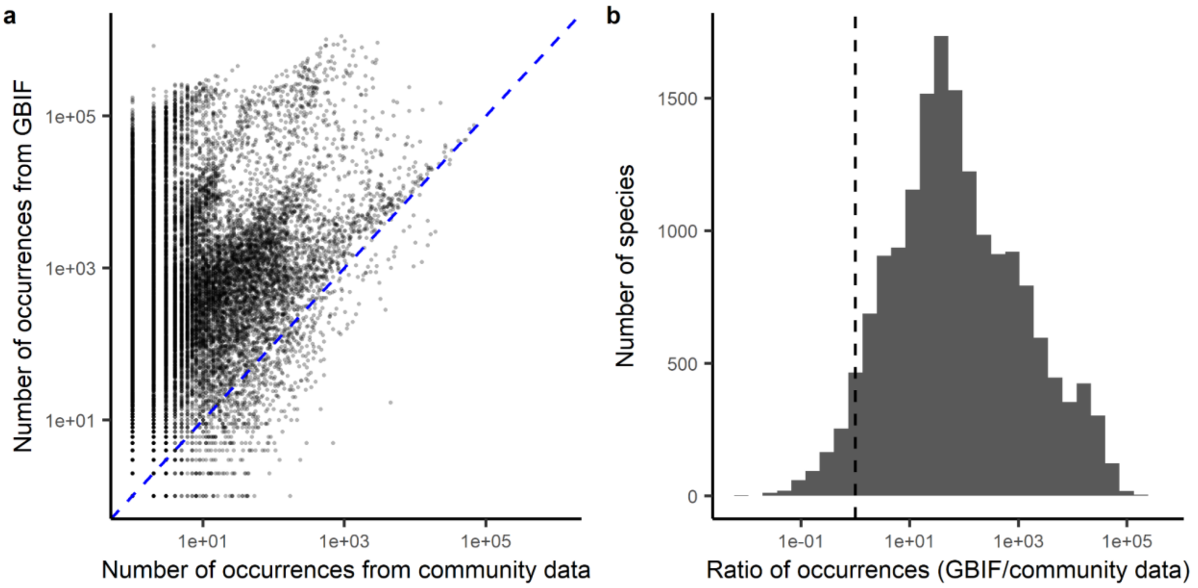

Supplementary Fig. 5 | Comparison of the number of occurrences from GBIF and assemblage datasets (a) and frequency distribution of the ratio between them (b). The number of occurrences was measured as the number of occupied grid-cells in 0.01°. The blue dashed line in panel **a** is the identity line. The vertical dashed line in panel **b** shows the same number of occurrences from GBIF and the assemblage dataset (*ratio* = 1).

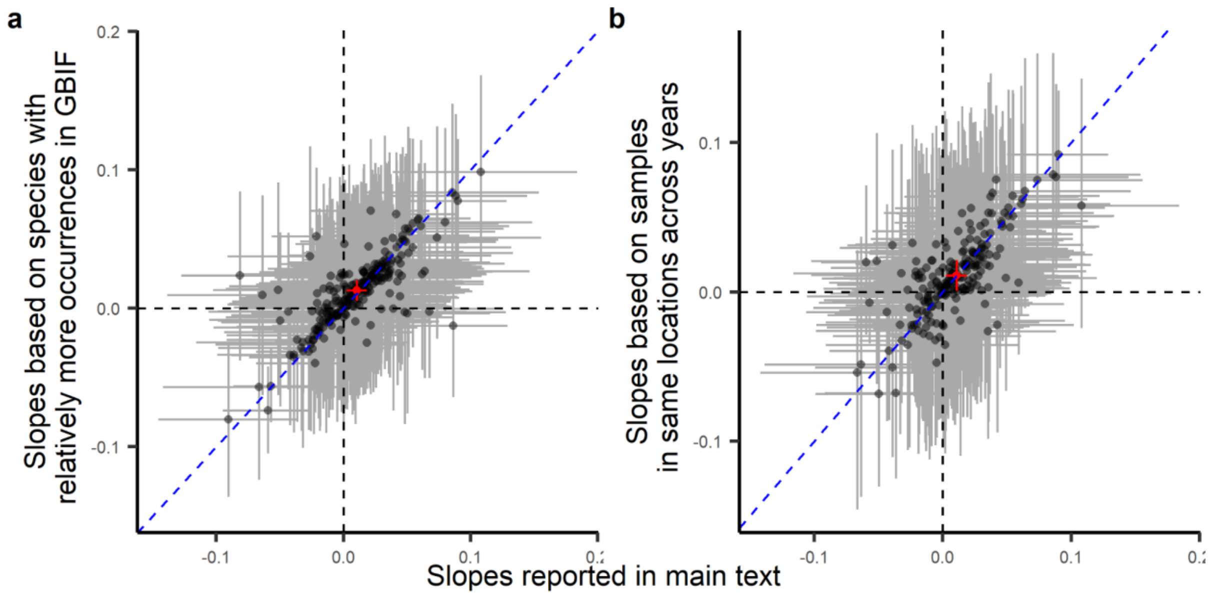

Supplementary Fig. 6 | Sensitivity analyses for using different data subsets. Comparison of the slopes reported in the main text (x-axis) with those obtained from analyses using data subsetted in different ways (y-axis). **a** Slopes in the y-axis were from an analysis including only the species that had at least five times more occurrences in GBIF than in the assemblage dataset (24,520 observations across 237 studies); **b** slopes in the y-axis were from an analysis using assemblage data with sites in the same locations across years (18,331 observations across 206 studies). Black points and gray lines indicate the study-level slope estimates and their 95% credible intervals; the red point and line indicate the overall slope estimate and its 95% credible interval. The blue dashed line indicates the identity line.

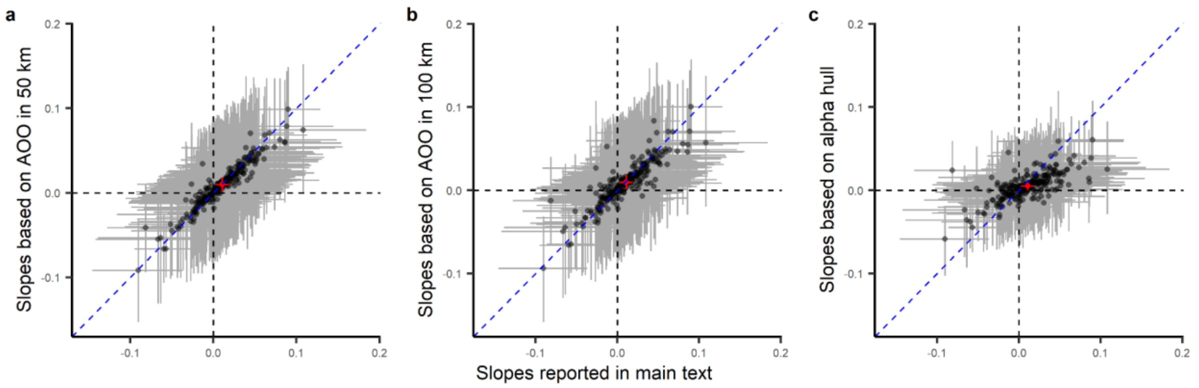

Supplementary Fig. 7 | Sensitivity analyses for using different estimates of range size. Comparison of the slopes reported in the main text (x-axis) with those obtained from analyses using different estimates of range size (y-axis). The main analyses used the area of occupancy (AOO) defined as the number of 10-km grid cells where distribution occurrences were observed. The sensitivity analyses used the AOO in the resolution of 50 km (**a**) and 100 km (**b**) and the area of alpha hulls (**c**). All analyses used 30,103 observations across 238 studies. Black points and gray lines indicate the study-level slope estimates and their 95% credible intervals; the red point and line indicate the overall slope estimate and its 95% credible interval. The blue dashed line indicates the identity line.

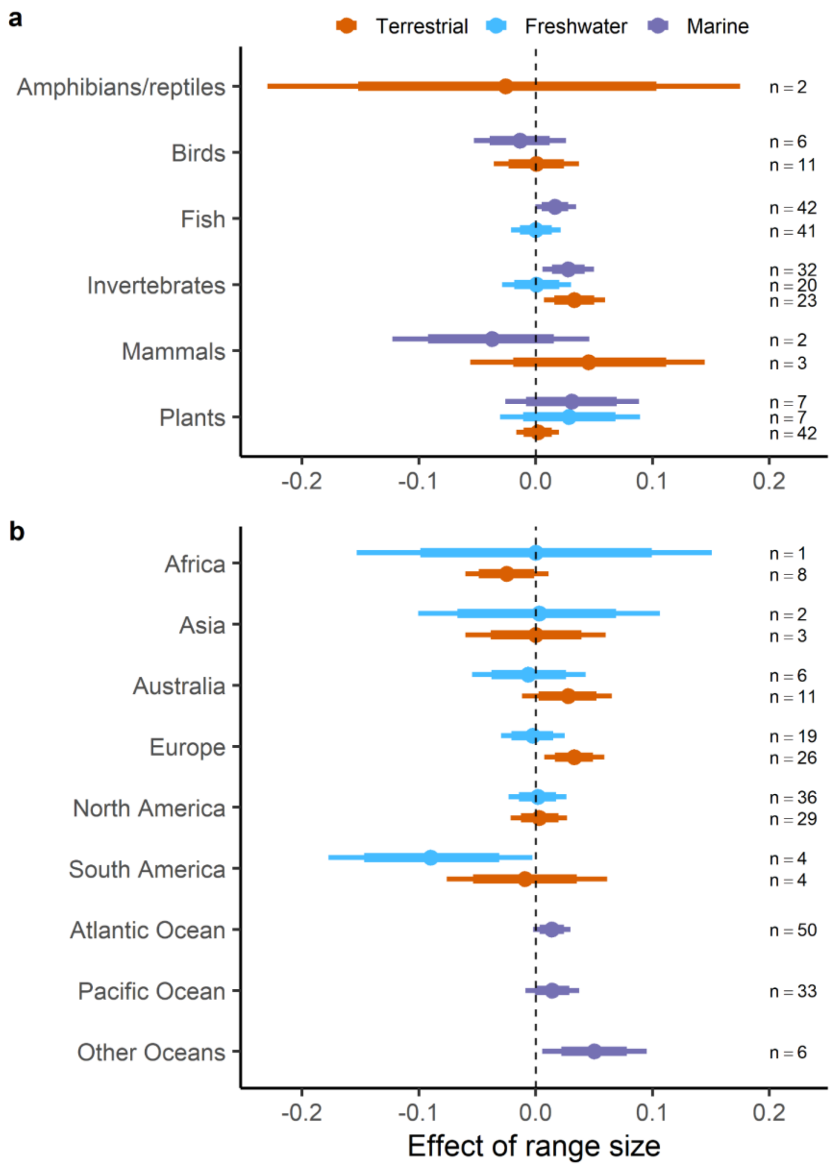

Supplementary Fig. 8 | Effect of range size on occupancy change across groups of studies. Studies were grouped by taxon group (**a**), and geographic region (continent and ocean; **b**) for terrestrial (orange), freshwater (blue) and marine (purple) realms. Mean (points), 95% (thick lines) and 80% (thin lines) credible intervals are shown for each group. The dashed line shows the zero slope (no effect of range size). The numbers on the right indicate the number of studies included in each group. We note that each study has at least 10 species-level observations (see Supplementary Table 5 for the number of species in each study), and we thus can estimate the uncertainty in the effects of range size even for groups with only one or two studies.

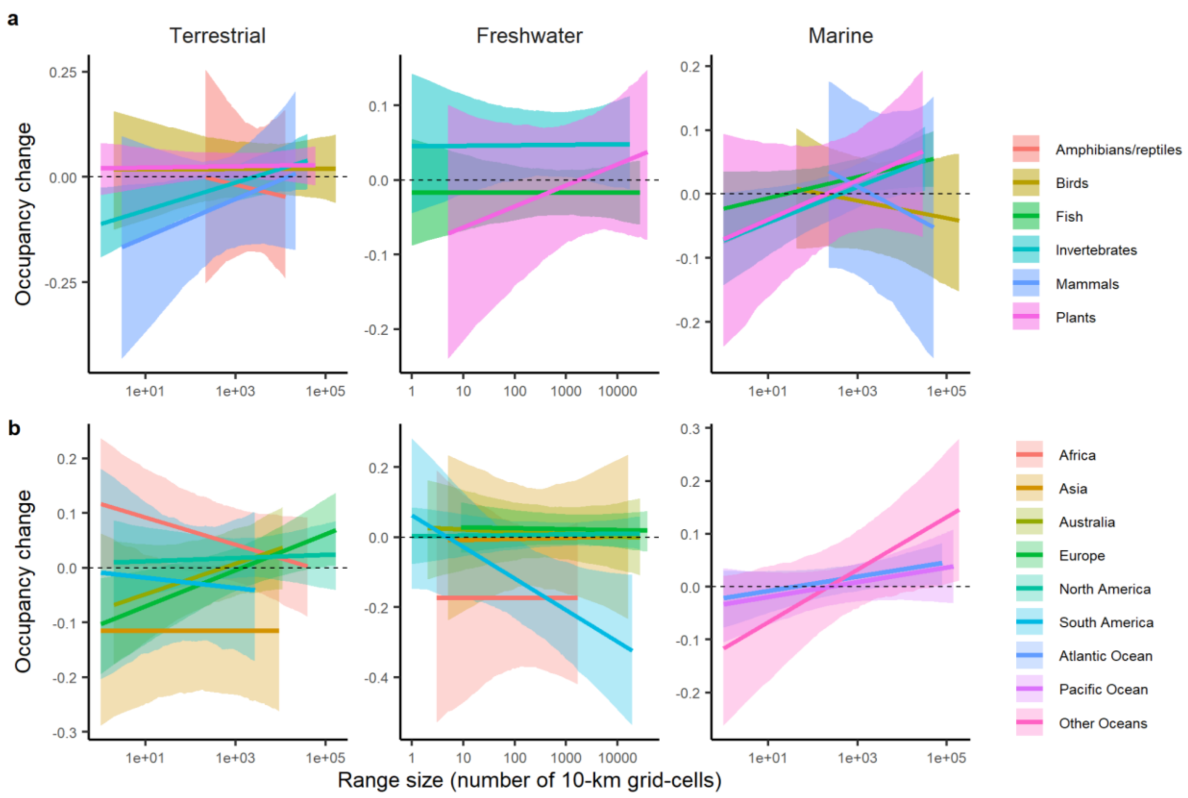

Supplementary Fig. 9 | The relationship between range size and occupancy change across groups of studies. Studies were grouped by taxon group (**a**), and geographic region (continent and ocean; **b**) for terrestrial, freshwater and marine realms. Occupancy change is the difference in occupancy between the late and early periods, shown as the square root transformed for the absolute magnitude. Solid lines and shading show the range size-occupancy change relationships and 95% credible intervals, respectively.

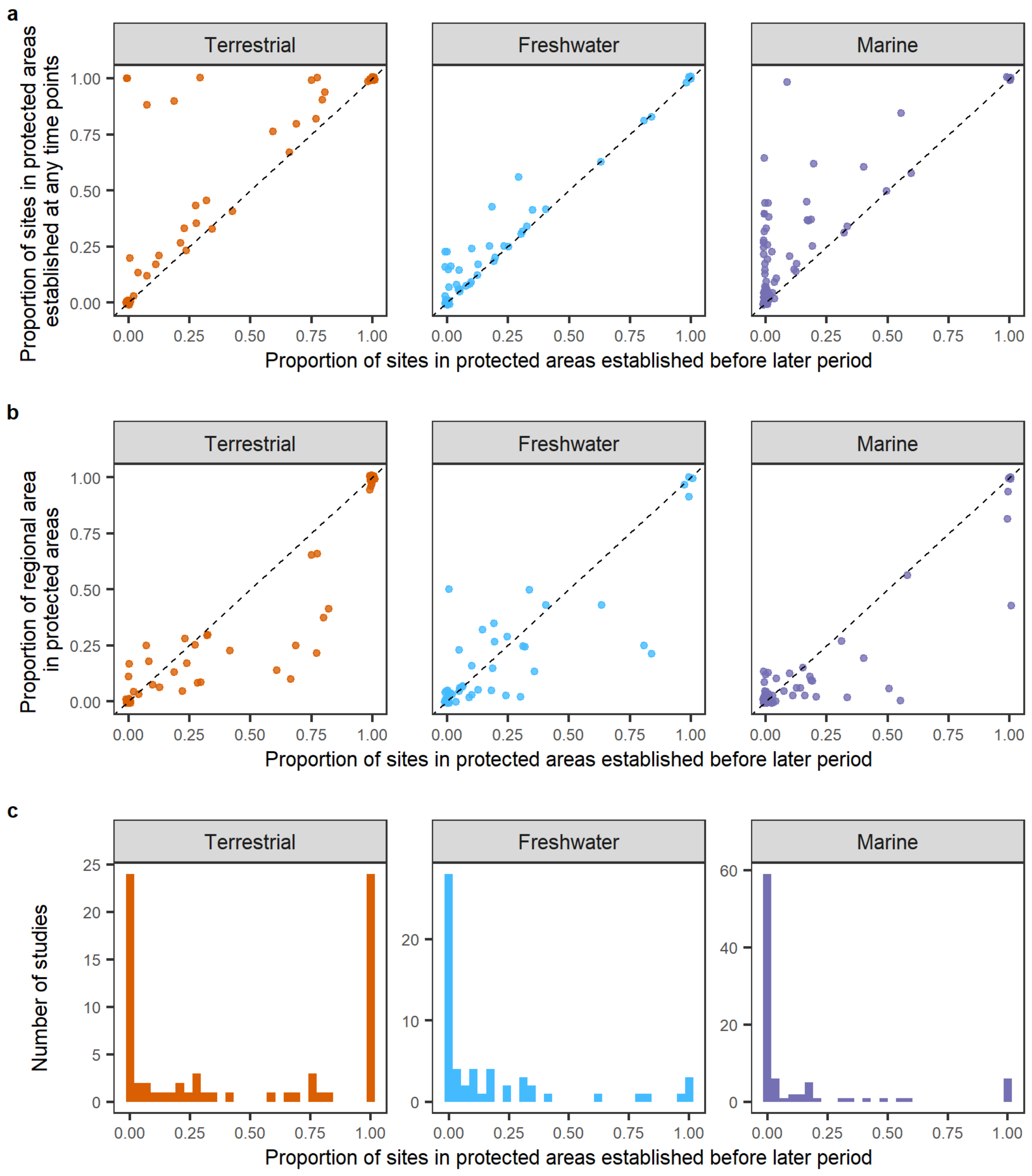

Supplementary Fig. 10 | Comparison of different estimates measuring the degree to which a metacommunity is protected. a Comparison of the proportion of sites in protected areas established at any time point and those that were established before the sampling in the late period. **b** Comparison of the proportion of local sites and the proportion of the spatial extent of each metacommunity in protected areas that were established before the sampling in the late period. **c** Frequency distribution of the proportion of sites in protected areas that were established before the sampling in the late period. The dashed lines in panels **a** and **b** are the identity line. The comparison was performed for terrestrial (orange), freshwater (blue) and marine (purple) realms, respectively.

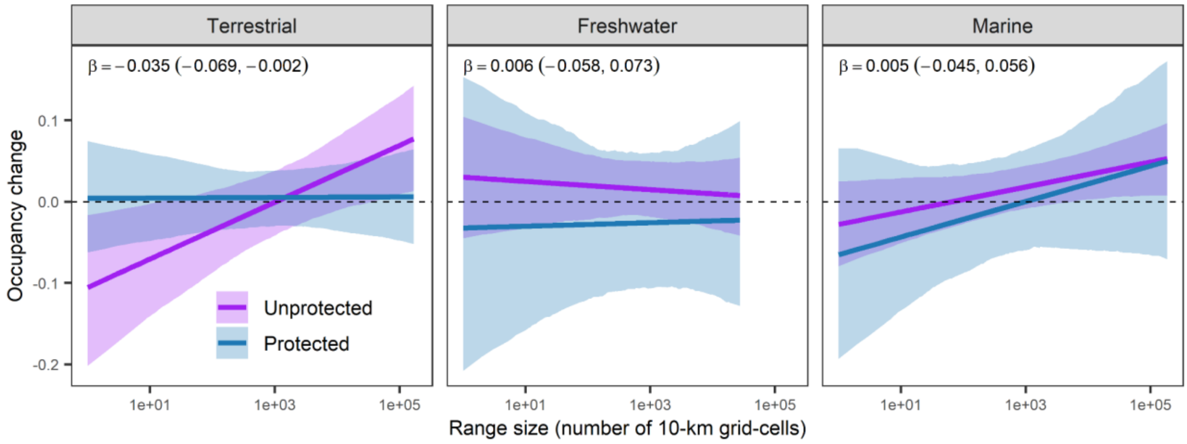

Supplementary Fig. 11 | Effect of habitat protection on the relationship between range size and occupancy change for terrestrial, freshwater and marine realms. This result was based on the proportion of sites in protected areas established at any time point, including those that were established more recently. Note that Fig. 3 shows results based on protected areas established before the sampling in the late period. The coefficient (β) of the interaction between range size and protection level for each realm and its 95% credible interval are shown at the top. The purple solid lines show the predicted relationship when no sites within a metacommunity are protected, while the blue solid lines show the predicted relationship when all sites within a metacommunity are protected; the shading shows the 95% credible intervals.

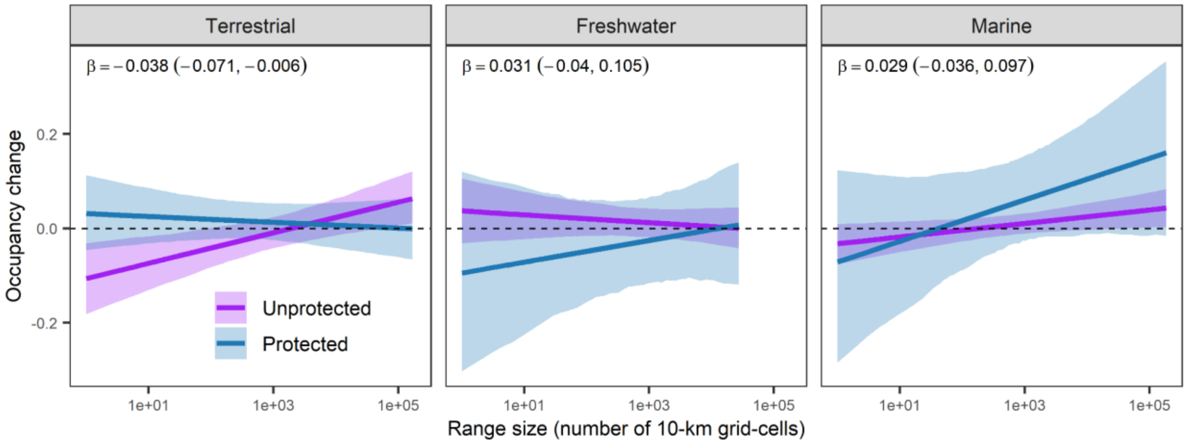

Supplementary Fig. 12 | Effect of habitat protection on the relationship between range size and occupancy change for terrestrial, freshwater and marine realms. This result was based on the proportion of the spatial extent of each metacommunity falling within protected areas that were established before the sampling in the late period. Note that Fig. 3 shows results based on the proportion of local sites in protected areas. The coefficient (β) of the interaction between range size and protection level for each realm and its 95% credible interval are shown at the top. The purple solid lines show the predicted relationship when no sites within a metacommunity are protected, while the blue solid lines show the predicted relationship when all sites within a metacommunity are protected; the shading shows the 95% credible intervals.

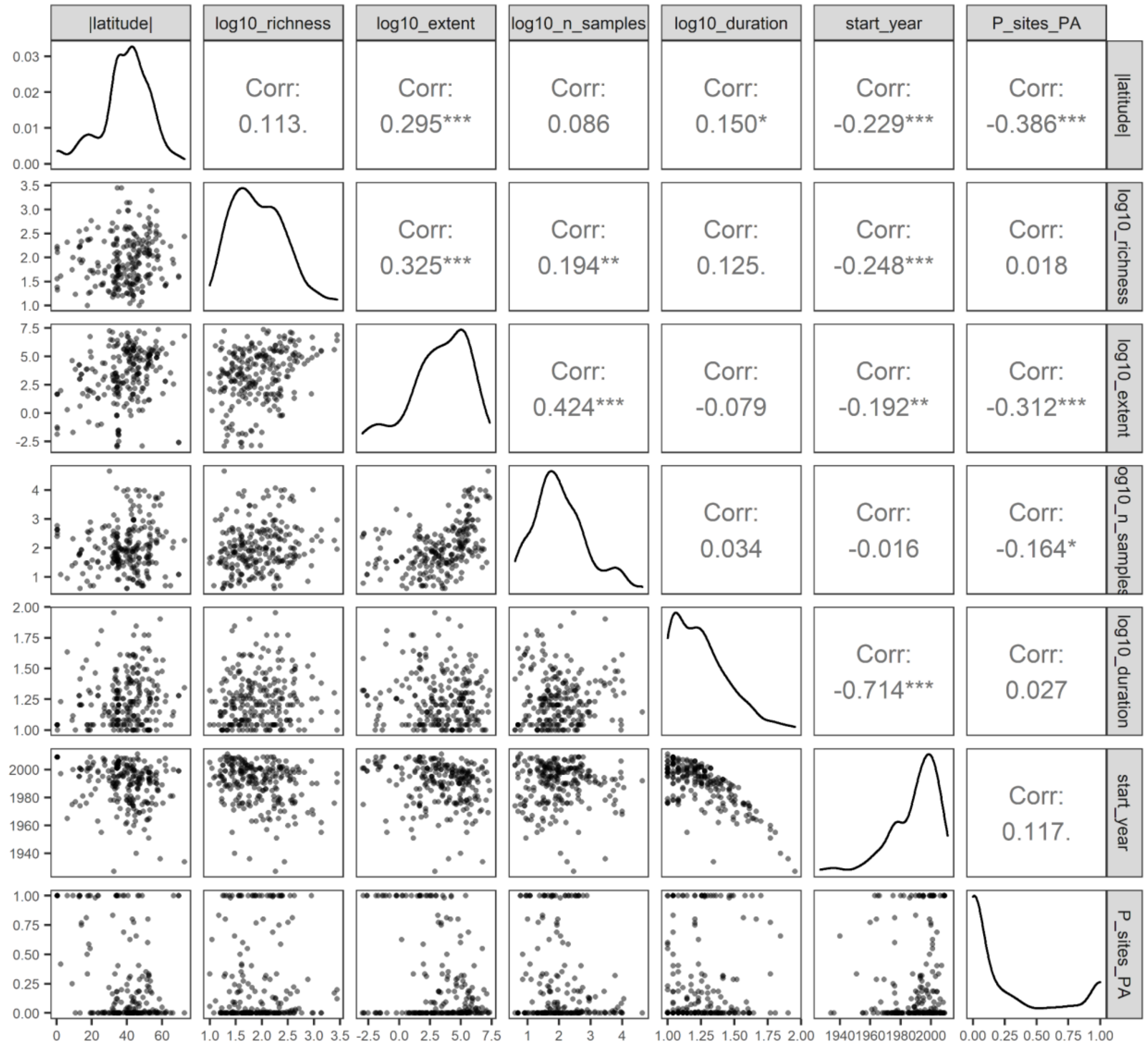

Supplementary Fig. 13 | Pearson correlations among study characteristics. The characteristics include absolute central latitude of study sites (|latitude|), regional species richness observed in the study (log_{10}-transformed, richness), extent of study sites (log_{10}-transformed, extent), number of samples used to calculate occupancy in each period (log_{10}-transformed, n_samples), duration of sampling (log_{10}-transformed, duration), start year of sampling (start_year), and proportion of sites in protected areas (P_sites_PA).

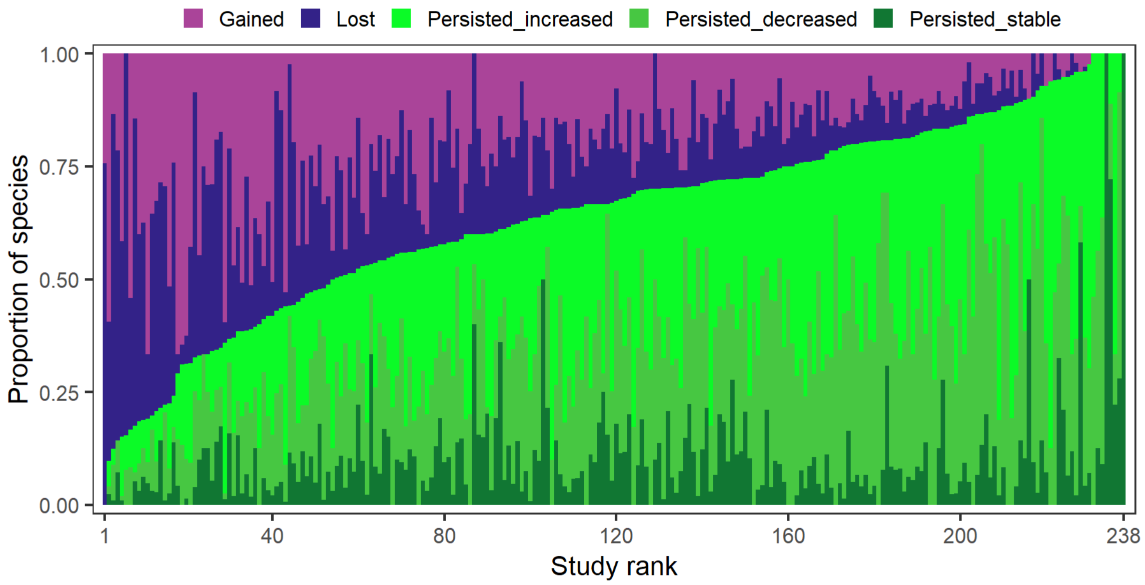

Supplementary Fig. 14 | Proportion of species experiencing different dynamics within studies. Dynamics of species were classified as gained (purple), lost (dark blue), persisted with increased occupancy (light green), decreased occupancy (green), and no changed (stable; dark green) occupancy. Each bar represents a study. The medians of the proportion of species in different dynamics are 0.167, 0.120, 0.265, 0.243, and 0.073, respectively. Fifty-four of 238 studies have gained and lost species accounting for more than 50% of total recorded species.

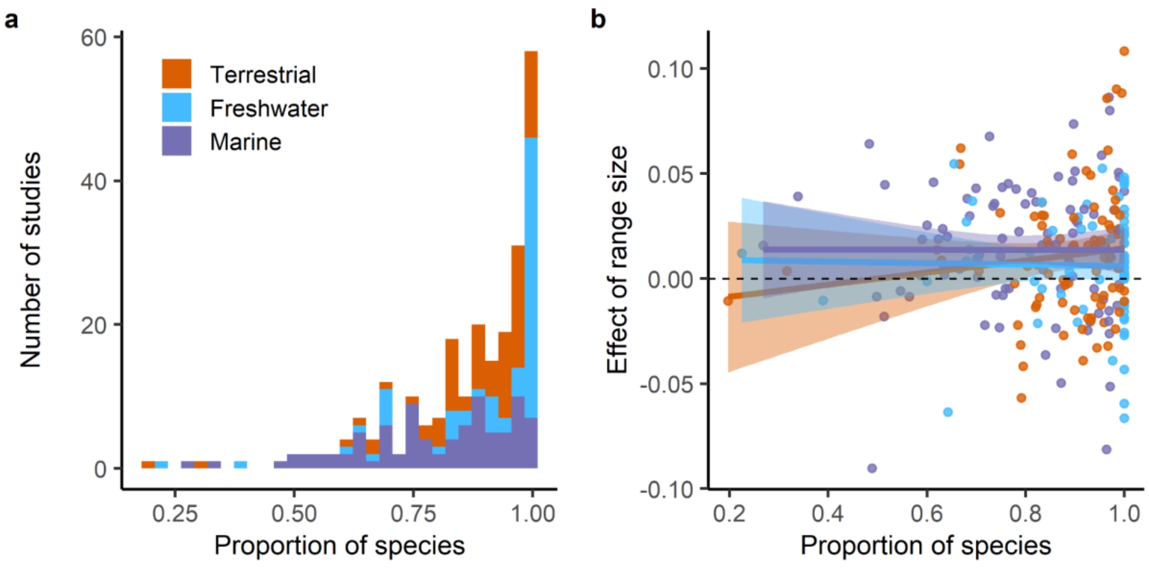

Supplementary Fig. 15 | Proportion of species within studies that have data on estimates of range size. (a) Frequency distribution of the proportion of species that have range size estimates and (**b**) its association with study-level effects of range size on occupancy change. Solid lines and shading in panel **b** show the linear relationship and 95% credible interval. The colors indicate the terrestrial (orange), freshwater (blue) and marine (purple) realms where studies come from. All linear relationships are not significant (two-sided *t*-test, *p*-value = 0.25, 0.88 and 0.99 for terrestrial, freshwater and marine realm, respectively).

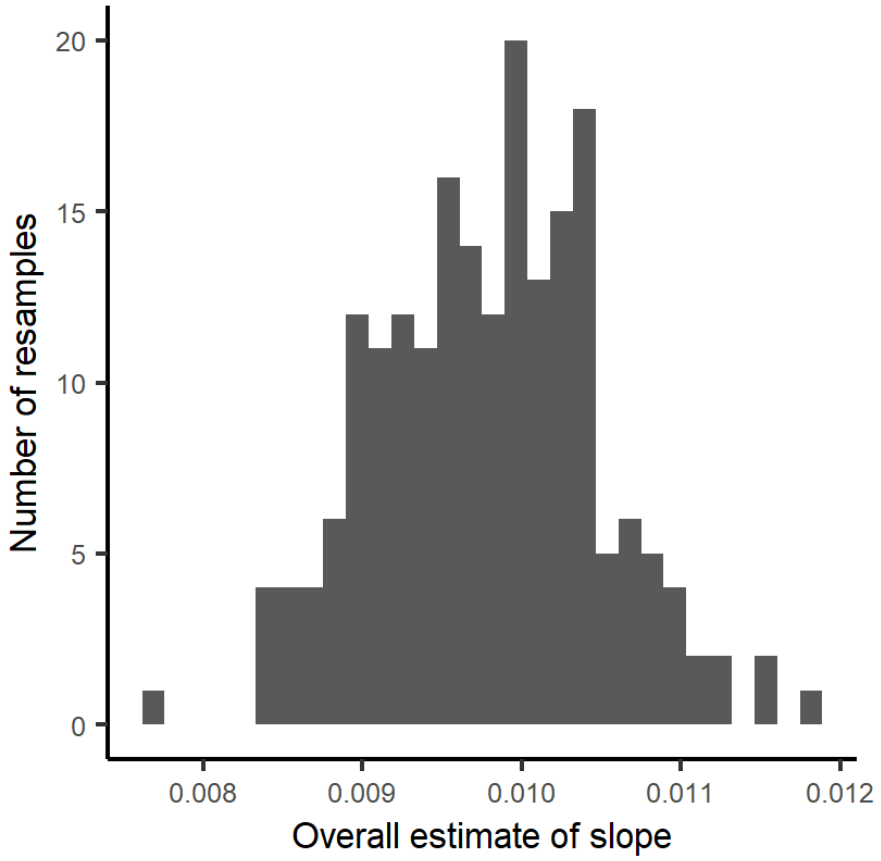

Supplementary Fig. 16 | Frequency distribution of the overall slope estimate of the relationship between range size and occupancy using datasets from 200 iterations of rarefaction.

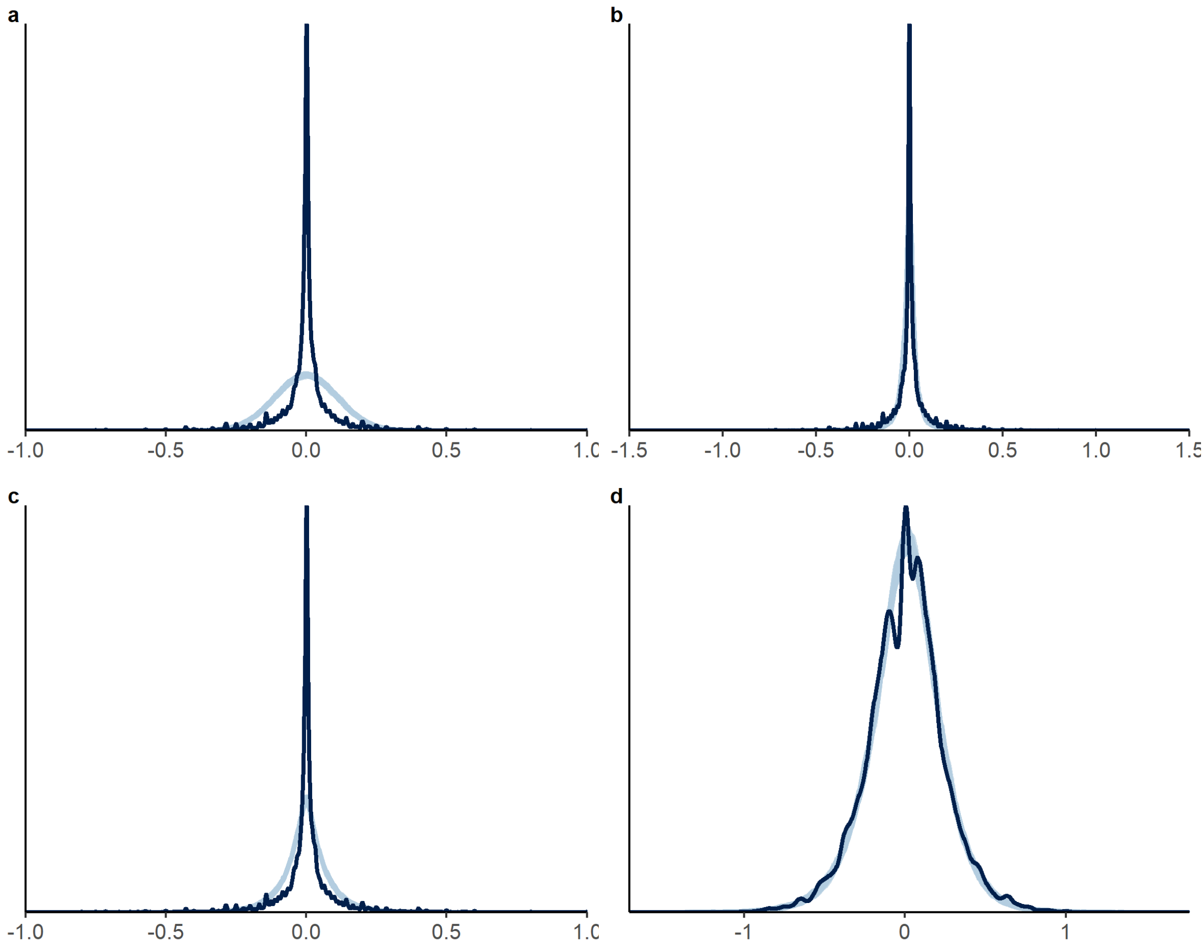

Supplementary Fig. 17 | Comparing kernel density estimates of the observed occupancy changes (black curves) and predicted occupancy changes (blue curves) drawn from the posterior predictive distribution. a-c Predicted occupancy changes came from models regressing occupancy change as a function of range size using Gaussian (**a**), Student's t (**b**), and asymmetric Laplacian (**c**) error distributions, respectively. **d** Predicted occupancy changes came from the model regressing sign*square-root-transformed occupancy change as a function of range size using Gaussian error distribution.

Supplementary Table 1 | Summary of the model testing the overall effect of range size on temporal occupancy change across 238 studies. Range size was log_{10}-transformed and centered before fitting models. n_samples was the log_{10}-transformed number of samples in each period, which was used to fit variation in the standard deviation of occupancy changes across studies. For each parameter, the mean estimate, the standard deviation (sd), and the 95% credible interval (Q2.5 and Q97.5) were shown. Rhat is the Gelman-Rubin convergence diagnostic; Bulk- and Tail-ESS are the number of independent samples (i.e., effective sample sizes). The model used 30,103 observations across 238 studies.

Parameter	Estimate	sd	Q2.5	Q97.5	Rhat	Bulk_ESS	Tail_ESS
Intercept	0.008	0.006	-0.005	0.020	1.00	1324	1819
sigma_Intercept	-0.680	0.011	-0.702	-0.658	1.00	4067	3849
Range	0.011	0.004	0.003	0.019	1.00	3318	3463
sigma_n_samples	-0.436	0.005	-0.445	-0.426	1.00	4034	3930

Supplementary Table 2 | Summary of the model testing the variation in effects of range size on temporal occupancy change among terrestrial, freshwater and marine realms. The model included interaction between range size and realm. Range size was log_{10}-transformed and centered before fitting models. n_samples was the log_{10}-transformed number of samples in each period, which was used to fit variation in the standard deviation of occupancy changes across studies. For each parameter, the mean estimate, the standard deviation (sd), and the 95% credible interval (Q2.5 and Q97.5) were shown. Rhat is the Gelman-Rubin convergence diagnostic; Bulk- and Tail-ESS are the number of independent samples (i.e., effective sample sizes). The model used 30,103 observations across 238 studies, with 11,714, 4,223 and 14,166 observations in 81, 68 and 89 terrestrial, freshwater and marine studies, respectively.

Parameter	Estimate	sd	Q2.5	Q97.5	Rhat	Bulk_ESS	Tail_ESS
sigma_Intercept	-0.680	0.011	-0.702	-0.658	1.00	3648	3471
Terrestrial	0.006	0.011	-0.017	0.028	1.00	2358	2956
Freshwater	0.004	0.012	-0.020	0.029	1.00	3033	3566
Marine	0.013	0.010	-0.007	0.033	1.00	1308	2036
Terrestrial:Range	0.012	0.007	-0.002	0.025	1.00	3753	3970
Freshwater:Range	0.000	0.009	-0.016	0.017	1.00	3400	3546
Marine:Range	0.017	0.006	0.004	0.029	1.00	3366	3175
sigma_n_samples	-0.436	0.005	-0.445	-0.426	1.00	3728	3809

Supplementary Table 3 | Summary of the model testing the effect of habit protection on the relationship between range size and temporal occupancy change for terrestrial, freshwater and marine realms, respectively. The model included interactions among range size, protection level and realm. The protection level was measured as the proportion of sites in protected areas that were established before the sampling in the late period. Range size was log_{10}-transformed and centered before fitting models. n_samples was the log_{10}-transformed number of samples in each period, which was used to fit variation in the standard deviation of occupancy changes across studies. For each parameter, the mean estimate, the standard deviation (sd), and the 95% credible interval (Q2.5 and Q97.5) were shown. Rhat is the Gelman-Rubin convergence diagnostic; Bulk- and Tail-ESS are the number of independent samples (i.e., effective sample sizes). The model used 25,603 observations across 218 studies, with 8,440, 3,072 and 14,091 observations in 72, 58 and 88 terrestrial, freshwater and marine studies, respectively.

Parameter	Estimate	sd	Q2.5	Q97.5	Rhat	Bulk_ESS	Tail_ESS
sigma_Intercept	-0.648	0.012	-0.671	-0.624	1.00	3559	3700
Terrestrial	-0.016	0.018	-0.053	0.019	1.00	2847	3764
Freshwater	0.013	0.016	-0.019	0.043	1.00	2916	3519
Marine	0.012	0.011	-0.012	0.034	1.00	1153	1849
Terrestrial:Range	0.035	0.012	0.012	0.059	1.00	3597	3921
Freshwater:Range	-0.006	0.011	-0.027	0.015	1.00	3981	3970
Marine:Range	0.015	0.007	0.001	0.028	1.00	2998	3629
Terrestrial:Protection	0.027	0.028	-0.026	0.081	1.00	2702	3132
Freshwater:Protection	-0.032	0.048	-0.126	0.062	1.00	3769	3680
Marine:Protection	-0.005	0.041	-0.084	0.076	1.00	2839	3529
Terrestrial:Range:Protection	-0.039	0.017	-0.073	-0.005	1.00	3543	3811
Freshwater:Range:Protection	0.014	0.034	-0.054	0.080	1.00	3911	3962
Marine:Range:Protection	0.015	0.029	-0.043	0.073	1.00	3940	4101
sigma_n_samples	-0.438	0.005	-0.448	-0.428	1.00	3609	3772

Supplementary Table 4 | Summary of the model testing the effects of range size, seven study characteristics (including protection level) and their interactions on temporal occupancy changes for terrestrial, freshwater and marine realms, respectively. These study characteristics include the proportion of sites in protected areas (protection), absolute central latitude of study sites (latitude), regional species richness (richness), duration of sampling (duration), start year of sampling (start_year), extent of study sites (extent), and number of samples used to calculate occupancy in each period (n_samples). The model included an interaction between the realm and all other variables. Range size, richness, duration, extent, and n_samples were $\log_{10}$-transformed. All explanatory variables except the protection variable were centered by subtracting the mean value. For each parameter, the mean estimate, the standard deviation (sd), and the 95% credible interval (Q2.5 and Q97.5) were shown. Rhat is the Gelman-Rubin convergence diagnostic; Bulk- and Tail-ESS are the number of independent samples (i.e., effective sample sizes). The parameters with 95% credible interval non-overlapped with zero were shown in bold. The model used 25,603 observations across 218 studies, with 8,440, 3,072 and 14,091 observations in 72, 58 and 88 terrestrial, freshwater and marine studies, respectively.

Parameter	Estimate	sd	Q2.5	Q97.5	Rhat	Bulk_ESS	Tail_ESS
**sigma_Intercept**	**-0.6482**	**0.0118**	**-0.6718**	**-0.6260**	1.00	4064	3850
Terrestrial	-0.0174	0.0191	-0.0537	0.0198	1.00	3179	3563
Freshwater	0.0345	0.0211	-0.0069	0.0755	1.00	3342	3702
Marine	0.0028	0.0146	-0.0255	0.0322	1.00	2726	3416
**Terrestrial:Range**	**0.0398**	**0.0127**	**0.0147**	**0.0650**	1.00	3727	3739
Freshwater:Range	0.0104	0.0134	-0.0152	0.0376	1.00	3486	3776
**Marine:Range**	**0.0279**	**0.0106**	**0.0073**	**0.0487**	1.00	3436	3663
Terrestrial:Protection	0.0190	0.0321	-0.0448	0.0811	1.00	3202	3637
Freshwater:Protection	-0.0518	0.0490	-0.1497	0.0448	1.00	3735	3777
Marine:Protection	0.0272	0.0449	-0.0608	0.1176	1.00	3278	3630
Terrestrial:Latitude	-0.0010	0.0009	-0.0027	0.0007	1.00	3513	3739
**Freshwater:Latitude**	**0.0031**	**0.0015**	**0.0001**	**0.0061**	1.00	3630	3845
Marine:Latitude	0.0013	0.0010	-0.0005	0.0032	1.00	2740	3249
Terrestrial:Richness	-0.0467	0.0309	-0.1067	0.0141	1.00	3264	3668
Freshwater:Richness	0.0407	0.0431	-0.0470	0.1215	1.00	3365	3158
Marine:Richness	0.0197	0.0217	-0.0228	0.0624	1.00	2787	3335
Terrestrial:Duration	-0.0355	0.1252	-0.2699	0.2141	1.00	3660	3692
Freshwater:Duration	0.0461	0.1172	-0.1849	0.2797	1.00	3590	3813
Marine:Duration	0.0812	0.0761	-0.0707	0.2295	1.00	2685	3603
Terrestrial:Start_year	-0.0004	0.0017	-0.0037	0.0028	1.00	3711	3727
Freshwater:Start_year	-0.0007	0.0017	-0.0041	0.0027	1.00	3794	4015
Marine:Start_year	0.0001	0.0011	-0.0019	0.0023	1.00	2769	3631

Parameter	Estimate	sd	Q2.5	Q97.5	Rhat	Bulk_ESS	Tail_ESS
Terrestrial:Extent	-0.0030	0.0060	-0.0143	0.0088	1.00	3278	3659
Freshwater:Extent	-0.0096	0.0108	-0.0306	0.0114	1.00	3418	3814
Marine:Extent	-0.0093	0.0062	-0.0218	0.0028	1.00	2893	3694
Terrestrial:n_samples	0.0101	0.0201	-0.0305	0.0493	1.00	3423	3800
Freshwater:n_samples	-0.0033	0.0304	-0.0616	0.0571	1.00	2836	3545
**Marine:n_samples**	**0.0295**	**0.0139**	**0.0026**	**0.0567**	1.00	2629	3631
**Terrestrial:Range:Protection**	**-0.0473**	**0.0217**	**-0.0900**	**-0.0050**	1.00	3713	3853
Freshwater:Range:Protection	0.0288	0.0360	-0.0419	0.0995	1.00	3744	3510
Marine:Range:Protection	0.0031	0.0320	-0.0603	0.0657	1.00	3779	3575
Terrestrial:Range:Latitude	-0.0005	0.0006	-0.0017	0.0007	1.00	3919	3712
Freshwater:Range:Latitude	-0.0019	0.0010	-0.0038	0.0001	1.00	3876	3971
Marine:Range:Latitude	-0.0007	0.0006	-0.0019	0.0005	1.00	3599	3569
Terrestrial:Range:Richness	0.0014	0.0189	-0.0373	0.0394	1.00	3656	3835
**Freshwater:Range:Richness**	**0.0540**	**0.0268**	**0.0007**	**0.1056**	1.00	3684	3546
Marine:Range:Richness	-0.0003	0.0133	-0.0260	0.0262	1.00	3356	3552
Terrestrial:Range:Duration	-0.0233	0.0793	-0.1833	0.1324	1.00	3644	4055
Freshwater:Range:Duration	-0.0743	0.0799	-0.2314	0.0824	1.00	3919	4059
Marine:Range:Duration	-0.0400	0.0459	-0.1335	0.0495	1.00	3529	3813
Terrestrial:Range:Start_year	-0.0010	0.0010	-0.0031	0.0010	1.00	3630	3833
Freshwater:Range:Start_year	-0.0012	0.0012	-0.0035	0.0012	1.00	3911	3738
**Marine:Range:Start_year**	**-0.0012**	**0.0006**	**-0.0024**	**-0.0001**	1.00	3604	3675
Terrestrial:Range:Extent	0.0019	0.0039	-0.0056	0.0095	1.00	3769	3571
Freshwater:Range:Extent	-0.0087	0.0073	-0.0228	0.0058	1.00	3837	3852
Marine:Range:Extent	-0.0055	0.0041	-0.0139	0.0025	1.00	3683	3617
Terrestrial:Range:n_samples	-0.0196	0.0121	-0.0429	0.0043	1.00	4015	3930
Freshwater:Range:n_samples	0.0246	0.0203	-0.0150	0.0637	1.00	3755	3739
Marine:Range:n_samples	-0.0133	0.0085	-0.0301	0.0033	1.00	3563	3776
**sigma_n_samples**	**-0.4374**	**0.0049**	**-0.4468**	**-0.4276**	1.00	**4065**	**3927**

Supplementary Table 5 | Details on the datasets used in this study.

Database	StudyID	Start year	End year	Nr yrs	Nr spp	Realm	Taxon	Region	Reference
bt	108	1985	2005	10	93	Marine	Birds	Indian Ocean	1
bt	††110	1976	1995	13	1889	Marine	Invertebrates	Atlantic	2
bt	112	1992	2005	14	17	Marine	Fish	Atlantic	3
bt	113	1971	1997	6	675	Marine	Invertebrates	Atlantic	4
bt	117	1963	2002	7	57	Marine	Invertebrates	Pacific	5
bt	119	1978	2009	32	171	Marine	Fish	Atlantic	6
bt	121	2001	2010	6	47	Marine	Fish	Pacific	7
bt	123	2000	2009	10	127	Marine	Fish	Atlantic	8
bt	125	1990	2000	9	77	Marine	Fish	Atlantic	9
bt	135	1977	1986	3	13	Marine	Fish	Atlantic	10
bt	147	1961	1993	16	372	Marine	Invertebrates	Pacific	11
bt	148	1981	2004	18	380	Marine	Fish	Pacific	12
bt	152	1979	1988	8	12	Marine	Invertebrates	Pacific	13
bt	††162	1991	2004	9	1390	Marine	Invertebrates	Atlantic	14
bt	††163	1993	2004	12	241	Marine	Fish	Pacific	15
bt	†166	1969	1987	9	101	Marine	Birds	Atlantic	16,17
bt	†169	1987	2006	18	152	Marine	Birds	Pacific	18-20
bt	171	1995	2007	13	20	Marine	Mammals	Atlantic	21
bt	†172	1998	2009	12	33	Marine	Mammals	Atlantic	22,23
bt	173	2000	2011	9	139	Marine	Invertebrates	Pacific	24
bt	176	1999	2010	12	165	Marine	Invertebrates	Atlantic	25
bt	178	1977	2007	14	307	Marine	Fish	Pacific	26
bt	180	1970	1994	24	269	Marine	Fish	Atlantic	27
bt	†182	1990	2009	19	32	Marine	Fish	Atlantic	28
bt	183	1999	2011	13	13	Marine	Invertebrates	Atlantic	29
bt	††187	1997	2006	5	1645	Marine	Invertebrates	Atlantic	30
bt	189	2001	2010	9	205	Marine	Fish	Atlantic	31
bt	190	2001	2010	10	201	Marine	Fish	Atlantic	32
bt	191	1955	1968	11	810	Marine	Invertebrates	Atlantic	33
bt	195	1978	2007	30	378	Terrestrial	Birds	North America	34
bt	††196	1976	2012	35	131	Marine	Invertebrates	Atlantic	35
bt	197	1985	2013	28	101	Marine	Fish	Atlantic	36
bt	198	1991	2013	22	55	Marine	Fish	Atlantic	37
bt	200	1962	1985	10	888	Marine	Invertebrates	Atlantic	33
bt	††204	1977	1997	6	155	Marine	Invertebrates	Atlantic	38

Database	StudyID	Start year	End year	Nr yrs	Nr spp	Realm	Taxon	Region	Reference
bt	206	1993	2008	16	107	Marine	Fish	Atlantic	39
bt	208	1997	2007	11	181	Marine	Fish	Atlantic	40
bt	209	1991	2007	17	129	Marine	Fish	Atlantic	41
bt	210	1975	2011	37	185	Marine	Fish	Atlantic	42
bt	††213	1968	2008	41	678	Marine	Fish	Atlantic	43
bt	214	1940	2009	42	29	Terrestrial	Plants	North America	44
bt	215	1985	2008	22	16	Terrestrial	Birds	North America	45
bt	217	1994	2004	7	205	Terrestrial	Birds	North America	46
bt	219	1995	2011	17	11	Terrestrial	Amphibians and reptiles	North America	47
bt	220	1995	2011	14	180	Terrestrial	Birds	North America	47
bt	229	1990	2013	19	61	Freshwater	Fish	North America	48
bt	232	1981	1993	7	68	Marine	Fish	Southern Ocean	49
bt	237	1976	1985	10	54	Freshwater	Invertebrates	North America	50
bt	244	1999	2012	14	142	Marine	Birds	Pacific	51
bt	247	1975	2006	31	18	Freshwater	Invertebrates	North America	52
bt	252	1978	1989	11	31	Marine	Fish	Atlantic	53
bt	253	1986	2014	29	72	Freshwater	Invertebrates	North America	54
bt	256	1996	2009	14	100	Marine	Fish	Atlantic	41
bt	271	2001	2014	14	54	Marine	Fish	Pacific	55
bt	272	2004	2014	11	32	Marine	Invertebrates	Pacific	56
bt	273	2004	2014	11	20	Marine	Invertebrates	Pacific	57
bt	†274	2001	2014	14	81	Marine	Invertebrates	Pacific	58
bt	278	1979	1990	7	216	Marine	Fish	Pacific	12
bt	286	2000	2009	9	18	Marine	Fish	Atlantic	8
bt	288	1972	1995	20	142	Marine	Fish	Atlantic	6
bt	290	1978	1988	4	170	Marine	Fish	Pacific	59
bt	291	1978	1988	2	349	Marine	Fish	Indian Ocean	59
bt	292	1978	1989	4	98	Marine	Fish	Pacific	59
bt	297	2005	2015	11	10	Marine	Invertebrates	Pacific	60
bt	300	1989	2013	25	14	Terrestrial	Invertebrates	North America	61
bt	304	1966	1996	2	418	Terrestrial	Plants	Africa	62-64
bt	309	1976	1985	10	42	Terrestrial	Invertebrates	Europe	65
bt	321	1995	2007	13	16	Terrestrial	Mammals	North America	66
bt	331	1998	2009	4	35	Terrestrial	Plants	North America	67-70
bt	354	1998	2013	4	174	Marine	Plants	Pacific	71
bt	356	1976	2012	21	372	Terrestrial	Plants	Australia	72

Database	StudyID	Start year	End year	Nr yrs	Nr spp	Realm	Taxon	Region	Reference
bt	359	2001	2012	12	46	Marine	Fish	Pacific	56
bt	374	2004	2014	11	61	Marine	Birds	Pacific	73
bt	375	2004	2014	11	115	Terrestrial	Invertebrates	Asia	74
bt	418	1995	2014	16	241	Marine	Fish	Pacific	75
bt	428	1936	2015	75	49	Marine	Fish	Atlantic	76-79
bt	430	1992	2016	22	47	Freshwater	Fish	Australia	80
bt	431	1997	2011	13	34	Freshwater	Fish	Australia	80
bt	432	1998	2012	14	25	Freshwater	Fish	Australia	80
bt	466	1986	2008	20	89	Marine	Fish	Atlantic	81
bt	467	1987	1999	4	71	Marine	Fish	Atlantic	82-85
bt	††468	1986	2011	21	73	Marine	Invertebrates	Pacific	86
bt	††469	1982	2011	30	37	Marine	Invertebrates	Pacific	87
bt	499	1985	1999	9	116	Marine	Invertebrates	Atlantic	88
bt	500	2001	2012	11	203	Marine	Invertebrates	Atlantic	89
bt	†505	1991	2011	7	164	Marine	Fish	Atlantic	90
bt	507	1988	2010	19	32	Marine	Fish	Atlantic	91
bt	512	1964	1994	2	136	Terrestrial	Plants	Europe	92,93
bt	516	1997	2013	4	41	Terrestrial	Mammals	South America	94-97
bt	525	1986	2014	4	25	Freshwater	Plants	Asia	98
bt	†527	2006	2015	10	95	Marine	Birds	Atlantic	99
bt	67	1994	2006	13	57	Terrestrial	Birds	Africa	100
bt	††78	1980	2004	20	192	Marine	Invertebrates	Atlantic	101
bt	84	1967	2005	5	95	Marine	Plants	Atlantic	102
bt	85	1992	2003	11	256	Marine	Invertebrates	Atlantic	103
bt	86	1986	1998	5	135	Marine	Plants	Atlantic	104
bt	90	1995	2004	3	21	Marine	Invertebrates	Atlantic	105
bt	††92	1981	1991	4	165	Marine	Invertebrates	Arctic Ocean	106
bt	97	1934	1955	2	140	Marine	Invertebrates	Arctic Ocean	107
bt	99	1982	1997	10	557	Marine	Fish	Indian Ocean	108
ft	1	2003	2017	3	27	Freshwater	Fish	Europe	109-111
ft	10	1988	2018	28	22	Freshwater	Fish	North America	112,113
ft	11	1988	2005	16	20	Freshwater	Fish	North America	114
ft	14	1999	2014	14	20	Freshwater	Fish	Asia	115
ft	15	2001	2017	5	93	Freshwater	Fish	North America	116
ft	16	1991	2008	2	34	Freshwater	Fish	Europe	117
ft	17	1975	1986	12	55	Freshwater	Fish	Africa	118
ft	18	1977	1988	6	57	Freshwater	Fish	North America	119

Database	StudyID	Start year	End year	Nr yrs	Nr spp	Realm	Taxon	Region	Reference
ft	2	2003	2013	2	53	Freshwater	Fish	South America	120,121
ft	20	1995	2013	15	56	Freshwater	Fish	North America	122
ft	21	1993	2018	26	115	Freshwater	Fish	North America	123
ft	23	2005	2019	8	30	Freshwater	Fish	Australia	124
ft	24	2008	2018	3	80	Freshwater	Fish	North America	125
ft	25	1989	2009	12	10	Freshwater	Fish	North America	126
ft	26	2002	2015	11	217	Freshwater	Fish	North America	127
ft	27	1993	2018	24	43	Freshwater	Fish	Europe	128
ft	28	1996	2016	7	123	Freshwater	Fish	North America	129
ft	29	2009	2019	3	19	Freshwater	Fish	South America	130
ft	30	1979	1994	15	83	Freshwater	Fish	North America	131,132
ft	32	1994	2017	24	74	Freshwater	Fish	Europe	133
ft	34	2002	2018	17	28	Freshwater	Fish	Europe	134
ft	36	1992	2018	20	30	Freshwater	Fish	North America	135
ft	37	1999	2012	2	19	Freshwater	Fish	South America	136
ft	39	2003	2015	5	48	Freshwater	Fish	North America	137
ft	4	2007	2018	11	41	Freshwater	Fish	Europe	138
ft	41	1990	2000	11	15	Freshwater	Fish	North America	139
ft	42	1989	2018	30	36	Freshwater	Fish	Europe	140
ft	43	2001	2017	17	11	Freshwater	Fish	Europe	141,142
ft	45	1995	2008	12	16	Freshwater	Fish	North America	143
ft	46	2001	2012	7	40	Freshwater	Fish	Europe	144,145
ft	5	1981	2002	21	57	Freshwater	Fish	North America	146
ft	6	2003	2018	16	53	Freshwater	Fish	Australia	147
ft	7	1999	2017	16	28	Freshwater	Fish	Europe	148
ft	9	2008	2018	9	27	Freshwater	Fish	North America	149
ic	1006	1993	2012	18	398	Terrestrial	Invertebrates	Europe	150
ic	1102	1965	2008	12	134	Terrestrial	Invertebrates	Europe	151
ic	1263	1994	2011	17	41	Terrestrial	Invertebrates	Europe	152
ic	1267	1994	2012	13	112	Terrestrial	Invertebrates	Europe	153
ic	1340	1969	2001	4	755	Terrestrial	Invertebrates	Europe	154
ic	1367	1980	2008	2	36	Terrestrial	Invertebrates	Europe	155
ic	1408	2000	2013	14	73	Freshwater	Invertebrates	Europe	156
ic	1444	1989	2008	17	22	Freshwater	Invertebrates	Australia	157
ic	1474	1951	2009	2	190	Terrestrial	Invertebrates	Europe	158
ic	1475	1964	2009	2	104	Terrestrial	Invertebrates	Europe	159
ic	1488	2006	2019	13	287	Freshwater	Invertebrates	Europe	160

Database	StudyID	Start year	End year	Nr yrs	Nr spp	Realm	Taxon	Region	Reference
ic	1520	1976	2015	36	38	Freshwater	Invertebrates	North America	161
ic	1525	2008	2019	12	29	Freshwater	Invertebrates	North America	162
ic	1526_1	2008	2018	10	19	Freshwater	Invertebrates	North America	163
ic	1526_2	2007	2020	9	38	Freshwater	Invertebrates	North America	164
ic	1533	1955	2018	2	17	Terrestrial	Invertebrates	South America	165
ic	1542	2002	2018	17	472	Freshwater	Invertebrates	Europe	166
ic	1547	2006	2017	8	820	Terrestrial	Invertebrates	Asia	167,168
ic	1553	1989	2004	15	29	Freshwater	Invertebrates	North America	169
ic	1554	2008	2018	11	38	Freshwater	Invertebrates	North America	170
ic	1555	2007	2019	13	16	Freshwater	Invertebrates	North America	171
ic	502	1971	1988	18	18	Terrestrial	Invertebrates	Europe	172
ic	79	1976	1985	9	36	Terrestrial	Invertebrates	Europe	172
mr	*sfd_11	2009	2019	9	23	Terrestrial	Plants	Africa	173,174
mr	sfd_12	2009	2018	8	20	Terrestrial	Plants	Africa	173,174
mr	sfd_13	2009	2019	9	33	Terrestrial	Plants	Africa	173,174
mr	sfd_131	2003	2012	2	15	Freshwater	Plants	North America	175,176
mr	sfd_156	1999	2017	8	38	Terrestrial	Plants	North America	177,178
mr	sfd_157	1999	2017	8	32	Terrestrial	Plants	North America	177,178
mr	sfd_158	2000	2015	4	389	Terrestrial	Plants	North America	179
mr	sfd_159	2001	2021	21	57	Marine	Fish	Pacific	180
mr	sfd_160	2001	2021	21	79	Marine	Invertebrates	Pacific	180
mr	sfd_161	2001	2021	21	26	Marine	Plants	Pacific	180
mr	sfd_163	1995	2013	3	312	Terrestrial	Plants	Australia	181
mr	sfd_164	2001	2016	4	155	Terrestrial	Plants	Australia	181
mr	sfd_165	2000	2015	4	193	Terrestrial	Plants	Australia	181
mr	sfd_166	1995	2013	3	158	Terrestrial	Plants	Australia	181
mr	sfd_167	2001	2016	4	86	Terrestrial	Plants	Australia	181
mr	sfd_168	2000	2015	4	74	Terrestrial	Plants	Australia	181
mr	sfd_169	1995	2012	3	58	Terrestrial	Birds	Europe	182
mr	sfd_17	2009	2019	11	134	Terrestrial	Plants	Africa	173,174
mr	sfd_170	1995	2012	5	66	Terrestrial	Birds	Europe	182
mr	sfd_172	2006	2020	12	187	Terrestrial	Plants	North America	183
mr	sfd_173	1974	2014	4	29	Marine	Plants	Atlantic	184
mr	sfd_174	1974	2014	4	19	Marine	Invertebrates	Atlantic	184
mr	sfd_175	2007	2017	2	166	Terrestrial	Plants	Europe	185
mr	sfd_176	1993	2018	4	14	Marine	Plants	Pacific	98

Database	StudyID	Start year	End year	Nr yrs	Nr spp	Realm	Taxon	Region	Reference
mr	sfd_178	1987	2020	4	24	Freshwater	Plants	North America	186
mr	sfd_179	2011	2020	2	18	Freshwater	Invertebrates	North America	186
mr	sfd_18	2009	2019	11	112	Terrestrial	Plants	Africa	173,174
mr	sfd_180	1963	2012	27	44	Marine	Invertebrates	Pacific	187
mr	sfd_183	1957	2015	2	122	Terrestrial	Plants	Europe	188
mr	sfd_186	1998	2016	15	133	Terrestrial	Plants	Australia	189
mr	sfd_187	1999	2017	7	46	Terrestrial	Mammals	Australia	190
mr	sfd_19	2009	2019	11	152	Terrestrial	Plants	Africa	173,174
mr	sfd_194	1992	2012	20	14	Terrestrial	Invertebrates	South America	191
mr	sfd_195	2002	2019	16	183	Terrestrial	Invertebrates	North America	192
mr	sfd_196	2002	2019	16	190	Terrestrial	Invertebrates	North America	192
mr	sfd_197	2002	2019	16	196	Terrestrial	Invertebrates	North America	192
mr	sfd_20	1999	2009	2	24	Marine	Invertebrates	Atlantic	193
mr	sfd_21	1974	1999	2	157	Marine	Fish	Pacific	194
mr	sfd_27	1997	2007	3	15	Marine	Plants	Pacific	195
mr	sfd_28	1981	2012	2	75	Marine	Fish	Pacific	196
mr	sfd_29	1990	2018	4	30	Freshwater	Invertebrates	North America	197
mr	sfd_30	1996	2009	2	16	Freshwater	Fish	North America	198
mr	sfd_31	1965	2007	2	139	Terrestrial	Invertebrates	Asia	199
mr	sfd_32	1927	2016	17	137	Terrestrial	Plants	North America	200
mr	sfd_33	1970	2015	2	218	Terrestrial	Plants	Europe	201
mr	sfd_34	1998	2007	2	351	Freshwater	Invertebrates	Europe	202-204
mr	sfd_35	1990	2007	3	308	Freshwater	Invertebrates	Europe	202-204
mr	sfd_36	1998	2007	2	180	Freshwater	Invertebrates	Europe	202-204
mr	sfd_37	1998	2007	2	107	Freshwater	Plants	Europe	205,206
mr	sfd_38	1998	2007	2	126	Freshwater	Plants	Europe	205,206
mr	sfd_39	1998	2007	2	59	Freshwater	Plants	Europe	205,206
mr	sfd_40	1990	2007	3	902	Terrestrial	Plants	Europe	207
mr	sfd_41	1990	2007	3	725	Terrestrial	Plants	Europe	207
mr	sfd_42	1998	2007	2	524	Terrestrial	Plants	Europe	207
mr	sfd_44	2009	2019	10	100	Terrestrial	Birds	North America	208
mr	sfd_47	1996	2011	6	247	Marine	Invertebrates	Atlantic	209
mr	sfd_48	1991	2006	3	102	Terrestrial	Birds	Europe	210
mr	sfd_49	1993	2009	7	76	Terrestrial	Birds	Europe	210
mr	sfd_50	1990	2011	4	111	Terrestrial	Birds	Europe	210
mr	sfd_51	1992	2013	5	13	Terrestrial	Invertebrates	Australia	211
mr	sfd_53	1975	2012	4	17	Marine	Fish	Pacific	212

Database	StudyID	Start year	End year	Nr yrs	Nr spp	Realm	Taxon	Region	Reference
mr	sfd_54	1990	2014	10	54	Terrestrial	Plants	Australia	213
mr	sfd_55	2008	2018	3	25	Terrestrial	Invertebrates	Europe	214
mr	sfd_56	2003	2019	2	24	Terrestrial	Invertebrates	Europe	214
mr	sfd_57	1976	2001	2	354	Terrestrial	Plants	North America	215
mr	sfd_58	1960	2018	2	314	Terrestrial	Plants	Europe	216
mr	sfd_59	1995	2004	10	14	Terrestrial	Amphibians and reptiles	North America	217
mr	sfd_60	2005	2015	2	45	Freshwater	Fish	South America	218
mr	sfd_62	1994	2004	2	10	Terrestrial	Plants	North America	219
mr	sfd_63	1972	2012	6	43	Freshwater	Fish	North America	220
mr	sfd_64	2000	2012	2	96	Terrestrial	Plants	South America	221
mr	sfd_65	2004	2021	9	44	Terrestrial	Plants	North America	222
mr	sfd_66	2004	2015	10	37	Terrestrial	Plants	North America	222
mr	sfd_67	2002	2016	14	54	Terrestrial	Plants	North America	222
mr	sfd_74	2006	2015	6	18	Terrestrial	Plants	North America	222
mr	sfd_75	2002	2021	19	51	Terrestrial	Plants	North America	222
mr	sfd_76	2006	2021	15	36	Terrestrial	Plants	North America	222
mr	sfd_78	2006	2021	14	20	Terrestrial	Plants	North America	222
mr	sfd_81	2006	2021	5	25	Terrestrial	Plants	North America	222
mr	sfd_82	1993	2015	20	32	Freshwater	Invertebrates	North America	223
mr	sfd_86	2003	2012	2	16	Freshwater	Plants	North America	175,176

Note: four databases were used: bt - BioTIME, ft - RivFishTIME, it - InsectChange, mr- Metacommunity Resurveys. † These studies were classified as 'multiple taxa' in the original data sources; †† These studies were classified as 'benthos' in the original data sources. * The prefix 'sfd' of StudyID indicates the self-defined ID in this study to keep the format of these datasets consistent with other datasets. Nr yrs: number of years that a given metacommunity was sampled; Nr spp: number of species with range size estimates in a given metacommunity.

Supplementary References

1. Australian Antarctic Data Centre. Seabirds of the Southern and South Indian Ocean. Occurrence dataset. GBIF.org https://doi.org/10.15468/tu0dcw (2022).
2. Network, N. B. Joint Nature Conservation Committee - Marine Nature Conservation Review (MNCR) and associated benthic marine data held and managed by JNCC. Occurrence dataset. GBIF.org https://doi.org/10.15468/ocjqwv (2012).
3. Ocean Biodiversity Information System. South Western Pacific Regional OBIS Data Asteroid Subset (South Western Paci fic OBIS). Occurrence dataset. GBIF.org https://doi.org/10.15468/notzfl (2012).
4. Pugh, P. Discovery Collections Midwater Database. National Oceanography Centre https://obis.org/dataset/2e9e9830-d7cf-43f3-ba38-12ac06a7c0ba (2000).
5. US National Oceanographic Data Center. South TX Outer Continental Shelf and MI, AL, and FL Outer Continental Shelf benthic organism sampling 1974-1978. US National Oceanographic Data Center https://obis.org/dataset/982d4bc2-c3f1-4018-befd-ed34b101e4b1 (2011).
6. Clark, D. & Branton, B. DFO Maritimes Research Vessel Trawl Surveys. OBIS Canada https://www.gbif.org/dataset/86711916-f762-11e1-a439-00145eb45e9a (2007).
7. Coral Reef Ecosystem Division (CRED), Pacific Island Fisheries Sciences Center and NOAA National Marine Fisheries Service. CRED Towed-Diver Fish Biomass Surveys in the Pacific Ocean 2000-2010. OBIS https://obis.org/dataset/1d3d2e69-3256-44c4-af75-ca6491f795c6 (2011).
8. Sherman, S. Maine Department of Marine Resources Inshore Trawl Survey, 2000-2009. Maine Department of Marine Resources https://www.usgs.gov/ocean-biodiversity-information-system-usa (2010).
9. Reichert, M. SCDNR/NOAA MARMAP Program, SCDNR MARMAP Aggregate Data Surveys, The Marine Resources Monitoring, Assessment, and Prediction (MARMAP) Program: MARMAP Chevron Trap Survey 1990-2009. Marine Resources Research Institute https://obis.org/dataset/d1dd65ec-fc75-480d-ab5d-4a4379603173 (2009).
10. Tropical and Subtropical Western South Atlantic OBIS. Previous Fisheries REVIZEE Program. v2.0. OBIS http://doi.org/10.25607/fkn6ys (2018).
11. Ocean Biodiversity Information System. South Western Pacific Regional OBIS Data Bryozoan Subset (South Western Pacific OBIS). Occurrence dataset. GBIF.org https://doi.org/10.15468/w2u8ek (2012).
12. Ocean Biodiversity Information System. South Western Pacific Regional OBIS Data provider for the NIWA Marine Biodata Information System. Occurrence Dataset. GBIF.org https://doi.org/10.15468/zuuiyu (2012).
13. Nishida, S. CMarZ (Census of Marine Zooplankton)-Asia Database. National Museum of Nature and Science, Japan https://www.godac.jamstec.go.jp/bismal/e/dataset/cmarz_asia (2013).
14. U.S. Environmental Protection Agency. Environmental Monitoring and Assessment Program (EMAP) Database. OBIS https://obis.org/dataset/41a50544-957c-4193-8bbf-25a25f749e85 (2012).
15. United States Geological Survey. NOAA AFSC North Pacific Groundfish Observer. GBIF.org https://doi.org/10.15468/bg83kc (2012).
16. Read, A. J., Halpin, P. N., Crowder, L. B., Best, B. D. & Fujioka, E. OBIS-SEAMAP: mapping marine mammals, birds and turtles. World Wide Web electronic publication http://seamap.env.duke.edu (2010).

17 Hyrenbach, D., Huettmann, F. & Chardine, J. PIROP Northwest Atlantic 1965-1992. OBIS-SEAMAP http://seamap.env.duke.edu/dataset/280 (2012).
18 Rintoul, C., Schlagenhauf-Langabeer, B., Hyrenbach, K., Morgan, K. & Sydeman, W. *Atlas of California Current marine birds and mammals: Version 1, Unpublished report*. (PRBO Conservation Science, Petaluma, California, 2006).
19 Yen, P. P., Sydeman, W. J. & Hyrenbach, K. D. Marine bird and cetacean associations with bathymetric habitats and shallow-water topographies: implications for trophic transfer and conservation. *J. Mar. Syst.* **50**, 79-99 (2004).
20 Yen, P., Sydeman, W., Bograd, S. & Hyrenbach, K. Spring-time distributions of migratory marine birds in the southern California Current: Oceanic eddy associations and coastal habitat hotspots over 17 years. *Deep Sea Research Part II: Topical Studies in Oceanography* **53**, 399-418 (2006).
21 Dunn, C. Bahamas Marine Mammal Research Organisation Opportunistic Sightings. OBIS-SEAMAP http://seamap.env.duke.edu/dataset/329 (2013).
22 Machete, M. POPA cetacean, seabird, and sea turtle sightings in the Azores area 1998-2015. http://seamap.env.duke.edu/dataset/704 (2016).
23 Morato, T. et al. Evidence of a seamount effect on aggregating visitors. *Mar. Ecol. Prog. Ser.* **357**, 23-32 (2008).
24 Japan Agency for Marine-Earth Science and Technology. Marine Biological Sample Database. OBIS Japan https://obis.org/node/0d07a0ea-9c75-48e8-b3fd-c28d653f4270 (2012).
25 Kennedy, M. K. & Spry, J. A. BioChem: Atlantic Zone Monitoring Program (AZMP) Maritimes Region zooplankton collection. OBIS https://obis.org/dataset/f9a6159e-d03b-45f2-a44a-eafb791e5987 (2011).
26 Boutillier, J. A. & PBS Shellfish Data Unit. Pacific Shrimp Trawl Survey. OBIS Canada Digital Collections, OBIS SEAMAP http://seamap.env.duke.edu/dataset/103150261 (2012).
27 Brown, S. K. R., Zwanenburg, K. & Branton, R. ECNASAP East Coast of North America Groundfish data, East Coast North America Strategic Assessment Project. OBIS http://ipt.iobis.org/obiscanada/resource?r=ecnasap (accessed 2012).
28 Wade, E. J. DFO Gulf Region Snow Crab Research Trawl Surveys. OBIS Canada Digital Collections https://obis.org/dataset/b2fe1445-9bb8-4849-962b-0696d0e3e29f (2011).
29 Tremblay, J. M. & Branton, B. DFO Maritimes Research Vessel Trawl Surveys Invertebrate Observations. OBIS Canada Digital Collections https://www.eurobis.org/imis?module=dataset&dasid=4131 (2007).
30 National Centers for Coastal Ocean Science (NCCOS). National Benthic Infaunal Database (NBID). NOAA National Centers for Environmental Information https://www.fisheries.noaa.gov/inport/item/38811 (2003).
31 National Centers for Coastal Ocean Science. St. John, USVI Fish Assessment and Monitoring Data (2002 - Present). NOAA National Centers for Environmental Information https://obis.org/dataset/a9114765-9e28-4a43-9987-4fd9f398748a (2007).
32 National Centers for Coastal Ocean Science. St. Croix, USVI Fish Assessment and Monitoring Data (2002 - Present). NOAA National Centers for Environmental Information https://www.fisheries.noaa.gov/inport/item/39197 (2007).
33 Northeast Fisheries Science Center, National Marine Fisheries Service, NOAA & U.S. Department of Commerce. NEFSC Benthic Database. OBIS https://obis.org/dataset/e7c86904-aac7-4a17-a895-99a54c430d80 (2010).
34 Pardieck, K. L., Ziolkowski Jr., D. J. and Hudson, M.-A. R. North American Breeding Bird Survey Dataset 1966 - 2014, version 2014.0. U.S. Geological Survey,

Patuxent Wildlife Research Center https://www.pwrc.usgs.gov/BBS/RawData/ (2015).
35 Moore, J. J. & Howson, C. M. Survey of the rocky shores in the region of Sullom Voe, Shetland, July 2013, A report to SOTEAG from Aquatic Survey & Monitoring Ltd. Cosheston, Pembrokeshire https://www.soteag.org.uk/environmental-monitoring/monitoring-reports/ (2014).
36 International Council for the Exploration of the Sea. ICES DATRAS: Scottish West Coast Survey For Commercial Fish Species 1985-2013. https://datras.ices.dk (2013).
37 International Council for the Exploration of the Sea. ICES DATRAS: Baltic International Trawl Survey. https://datras.ices.dk/Data_products/Download/Download_Data_public.aspx (2013).
38 Degraer, S. et al. Macrobel: Long term trends in the macrobenthos of the Belgian Continental Shelf. GBIF.org https://www.gbif.org/en/dataset/83c25cb6-f762-11e1-a439-00145eb45e9a (2006).
39 International Council for the Exploration of the Sea. ICES DATRAS: Northern Irish Ground Fish Survey https://datras.ices.dk/Data_products/Download/Download_Data_public.aspx (2010).
40 International Council for the Exploration of the Sea. ICES DATRAS: French Southern Atlantic Bottom Trawl Survey. https://datras.ices.dk/Data_products/Download/Download_Data_public.aspx (2010).
41 International Council for the Exploration of the Sea. ICES DATRAS: Beam Trawl Survey. https://datras.ices.dk/Data_products/Download/Download_Data_public.aspx (2010).
42 International Council for the Exploration of the Sea. ICES DATRAS: North Sea International Bottom Trawl Survey https://datras.ices.dk/Data_products/Download/Download_Data_public.aspx (2010).
43 NOAA National Marine Fisheries Service (NMFS) & Northeast Fisheries Science Center. Northeast Fisheries Science Center Bottom Trawl Survey Data. OBIS-USA https://doi.org/10.15468/8c5pvg (2005).
44 Andrews Forest LTER Site & Franklin, J. F. Long-term growth, mortality and regeneration of trees in permanent vegetation plots in the Pacific Northwest, 1910 to present. Environmental Data Initiative https://doi.org/10.6073/pasta/2315afa15ad0a2317b49565da6258c47 (2012).
45 HMANA. Hawk Migration Association of North America (HMANA) https://www.hmana.org/ (2016).
46 Hutto, R. L. Northern Region Landbird Monitoring Program: a program designed to monitor more than long-term population trends. USDA Forest Service General Technical Report PSW-GTR-191 https://www.fs.usda.gov/treesearch/pubs/32091 (2005).
47 Birds Canada. Marsh Monitoring Program-Bird surveys. NatureCounts, a node of the Avian Knowledge Network, Birds Canada https://www.naturecounts.ca/ (2012).
48 McLarney, W. O., Meador, J. & Chamblee, J. F. Upper Little Tennessee River Biomonitoring Program Database. Coweeta Long Term Ecological Research Program https://biotime.st-andrews.ac.uk/getDatasetsQ.php?study=229 (2010).
49 Australian Antarctic Data Centre. Pelagic Fish Observations 1968-1999. Occurrence dataset. GBIF.org https://doi.org/10.15468/qqu2dp (2016).
50 Lathrop, R. Madison Wisconsin Lakes Zooplankton 1976 - 1994. Environmental Data Initiative https://doi.org/10.6073/pasta/ec3d0186753985147d4f283252388e05 (2000).
51 Birds Canada. British Columbia Coastal Waterbird Survey. NatureCounts, a node of the Avian Knowledge Network, Birds Canada https://www.naturecounts.ca/ (2004).

52 Rudstam, L. G. Zooplankton survey of Oneida Lake New York 1964 to 2012. KNB Data Repository https://knb.ecoinformatics.org/view/kgordon.17.56 (216).
53 Reichert, M. MARMAP Blackfish Trap Survey 1990-2009. OBIS https://obis.org/dataset/0d4d1d93-df06-4a12-8d03-bd1083864563 (2009).
54 NTL Long-Term Ecological Research Network. North Temperate Lakes LTER: Zooplankton - Trout Lake Area 1982 - current. Environmental Data Initiative https://lter.limnology.wisc.edu/node/55119 (2010).
55 Santa Barbara Coastal LTER & Reed, D. C. SBC LTER: Reef: Kelp forest community dynamics: Abundance and size of giant kelp (Macrocystis pyrifera), ongoing since 2000, ver 18. Environmental Data Initiative https://doi.org/10.6073/pasta/d90872297e30026b263a119d4f5bca9f (2014).
56 Santa Barbara Coastal LTER & Reed, D. C. SBC LTER: Reef: Kelp Forest Community Dynamics: Fish abundance, ver 28. Environmental Data Initiative https://doi.org/10.6073/pasta/e37ed29111b2fddffc08355252b8b8c7 (2014).
57 Santa Barbara Coastal LTER & Reed, D. C. SBC LTER: Reef: Kelp forest community dynamics: Invertebrate and algal density, ver 21. Environmental Data Initiative https://doi.org/10.6073/pasta/cd4cf864efecd69891dfe1d73b9ac9c3 (2014).
58 Santa Barbara Coastal LTER & Reed, D. C. SBC LTER: Reef: Kelp forest community dynamics: Cover of sessile organisms, Uniform Point Contact, ver 23. Environmental Data Initiative https://doi.org/10.6073/pasta/f906c91e98c2a5fe752dfa0ccdc8895f (2014).
59 CMAR. CSIRO Marine Data Warehouse. OBIS Australia https://doi.org/10.15468/asbeok (2012).
60 Moorea Coral Reef LTER & Carpenter, R. Coral Reef: Long-term Population and Community Dynamics: Other Benthic Invertebrates, ongoing since 2005 ver 29. Environmental Data Initiative https://doi.org/10.6073/pasta/61cc126fc5a5a0a99b62b3aaac56f3e8 (2015).
61 Landis, D. & Gage, S. Insect Populations via Sticky Traps at KBS-LTER. Environmental Data Initiative https://doi.org/10.6073/pasta/6b6cc0ad7897d9008e8cf918bbf552d2 (2014).
62 Privett, S. D. J., Cowling, R. M. & Taylor, H. C. Thirty years of change in the fynbos vegetation of the Cape of Good Hope Nature Reserve, South Africa. *Bothalia* **31**, 99-115 (2001).
63 Taylor, H. A vegetation survey of the Cape of Good Hope Nature Reserve. I. The use of association-analysis and Braun-Blanquet methods. *Bothalia* **15**, 245-258 (1984).
64 Thuiller, W., Slingsby, J. A., Privett, S. D. J. & Cowling, R. M. Stochastic species turnover and stable coexistence in a species-rich, fire-prone plant community. *PLoS One* **2**, e938 (2007).
65 Pollard, E., Hall, M. L. & Bibby, T. J. *Monitoring the abundance of butterflies 1976-1985*. (Nature Conservancy Council, Peterborough, 1986).
66 Bestelmeyer, B., Lightfoot, D. & Schooley, R. L. Rodent data from trapping webs in the long-term Small Mammal Exclusion Study (SMES) at Jornada Basin LTER, 1995-2007. Environmental Data Initiative https://doi.org/10.6073/pasta/0c38baecb2e10b4fe70d187ba6f08dda (2016).
67 Halpern, C. B., McKenzie, D., Evans, S. A. & Maguire, D. A. Initial responses of forest understories to varying levels and patterns of green-tree retention. *Ecol. Appl.* **15**, 175-195 (2005).
68 Halpern, C. B. & McKenzie, D. Disturbance and post-harvest ground conditions in a structural retention experiment. *Forest. Ecol. Manag.* **154**, 215-225 (2001).

69 Halpern, C. B., Halaj, J., Evans, S. A. & Dovciak, M. Level and pattern of overstory retention interact to shape long-term responses of understories to timber harvest. *Ecol. Appl.* **22**, 2049-2064 (2012).
70 Halpern, C. DEMO: Vegetation Data—Post-Harvest. Demonstration of Ecosystem Management Options. Forest Science Data Bank, Corvallis http://andlter.forestry.oregonstate.edu/data/abstract.aspx?dbcode=TP104 (2015).
71 Davies, C. H. et al. A database of marine phytoplankton abundance, biomass and species composition in Australian waters. *Sci Data* **3**, 160043 (2016).
72 Bradford, M. G., Murphy, H. T., Ford, A. J., Hogan, D. L. & Metcalfe, D. J. Long - term stem inventory data from tropical rain forest plots in Australia. *Ecology* **95**, 2362 (2014).
73 Monitoring Site 1000 Project & Biodiversity Center and Ministry of Environment of Japan. Monitoring site 1000 Shorebird Survey. http://www.biodic.go.jp/moni1000/findings/data/index.html (2013).
74 Monitoring Site 1000 Project & Biodiversity Center and Ministry of Environment of Japan. Monitoring site 1000 Forest and grassland research - Surface wandering beetles survey data. http://www.biodic.go.jp/moni1000/findings/data/index.html (2014).
75 Sweatman, H. P. A. et al. *Long-Term Monitoring of the Great Barrier Reef. Status Report Number 5. LTM No.5.* (Australian Institute of Marine Science, 2001).
76 Olsen, E. M., Carlson, S. M., Gjosaeter, J. & Stenseth, N. C. Nine decades of decreasing phenotypic variability in Atlantic cod. *Ecol. Lett.* **12**, 622-631 (2009).
77 Rogers, L. A. et al. Climate and population density drive changes in cod body size throughout a century on the Norwegian coast. *Proc. Natl. Acad. Sci. U.S.A.* **108**, 1961-1966 (2011).
78 Stenseth, N. C. et al. Dynamics of coastal cod populations: intra- and intercohort density dependence and stochastic processes. *P Roy Soc B-Biol Sci* **266**, 1645-1654 (1999).
79 Barcelo, C., Ciannelli, L., Olsen, E. M., Johannessen, T. & Knutsen, H. Eight decades of sampling reveal a contemporary novel fish assemblage in coastal nursery habitats. *Glob. Change Biol.* **22**, 1155-1167 (2016).
80 Stoffels, R. New Zealand Freshwater Fish Database (extended). The National Institute of Water and Atmospheric Research (NIWA), https://doi.org/10.15468/jbpw92 (2022).
81 Neat, F. & Campbell, N. Demersal fish diversity of the isolated Rockall plateau compared with the adjacent west coast shelf of Scotland. *Biol. J. Linn. Soc.* **104**, 138-147 (2011).
82 Pombo, L. & Rebelo, J. E. Spatial and temporal organization of a coastal lagoon fish community - Ria de Aveiro, Portugal. *Cybium* **26**, 185-196 (2002).
83 Pombo, L., Elliott, M. & Rebelo, J. E. Environmental influences on fish assemblage distribution of an estuarine coastal lagoon, Ria de Aveiro (Portugal). *Sci Mar* **69**, 143-159 (2005).
84 Pombo, L., Rebelo, J. E. & Elliott, M. The structure, diversity and somatic production of the fish community in an estuarine coastal lagoon, Ria de Aveiro (Portugal). *Hydrobiologia* **587**, 253-268 (2007).
85 Rebelo, J. E. The Ichthyofauna and Abiotic Hydrological Environment of the Ria-De-Aveiro, Portugal. *Estuaries* **15**, 403-413 (1992).
86 Kenner, M. C. et al. A multi-decade time series of kelp forest community structure at San Nicolas Island, California. *Ecology* **94**, 2654 (2013).

87 Kushner, D. J., Rassweiler, A., McLaughlin, J. P. & Lafferty, K. D. A multi-decade time series of kelp forest community structure at the California Channel Islands. *Ecology* **94**, 2655 (2013).
88 Institute of Agricultural & Fisheries research (ILVO). Macrobenthos monitoring at long-term monitoring stations in the Belgian part of the North Sea between 1979 and 1999. https://doi.org/10.14284/201 (2016).
89 Bio-environmental research group, Institute of Agricultural & Fisheries research (ILVO). Macrobenthos monitoring at long-term monitoring stations in the Belgian part of the North Sea from 2001 on. https://doi.org/10.14284/202 (2016).
90 Edelist, D., Rilov, G., Golani, D., Carlton, J. T. & Spanier, E. Restructuring the Sea: profound shifts in the world's most invaded marine ecosystem. *Divers. Distrib.* **19**, 69-77 (2013).
91 van Rijn, I. Trawl fisheries in the Israeli Mediterranean 1980-2013. BioTIME http://biotime.st-andrews.ac.uk (2016).
92 Hundt, R. *Ökologisch-geobotanische Untersuchungen an den mitteldeutschen Wiesengesellschaften unter besonderer Berücksichtigung ihres Wasserhaushaltes und ihrer Veränderung durch die Intensivbewirtschaftung im Rahmen der Großflächenproduktion*. Vol. 3 366 (Biosphärenreservat Rhön, Thüringen, 2001).
93 Jandt, U. & Bruelheide, H. German vegetation reference database (GVRD). *Biodiversity & Ecology* **4**, 355 (2012).
94 Rocha, R. et al. Consequences of a large-scale fragmentation experiment for Neotropical bats: disentangling the relative importance of local and landscape-scale effects. *Landsc. Ecol.* **32**, 31-45 (2017).
95 Rocha, R. et al. Secondary forest regeneration benefits old-growth specialist bats in a fragmented tropical landscape. *Sci. Rep.* **8**, 3819 (2018).
96 Sampaio, E. M., Kalko, E. K. V., Bernard, E., Rodriguez-Herrera, B. & Handley, C. O. A biodiversity assessment of bats (Chiroptera) in a tropical lowland rainforest of Central Amazonia, including methodological and conservation considerations. *Stud. Neotrop. Fauna Environ.* **38**, 17-31 (2003).
97 Rocha, J. R. T. *Tropical forest fragmentation: effects on the spatio-temporal dynamics of its bat communities*. (Universidade de Lisboa, Lisbon, Portugal, 2017).
98 Sarker, S. K. et al. 1980s-2010s: The world's largest mangrove ecosystem is becoming homogeneous. *Biol. Conserv.* **236**, 79-91 (2019).
99 Gjerdrum, C. & Fifield, D. CWS-EC Eastern Canada Seabirds at Sea (ECSAS). OBIS Canada Digital Collections https://obis.org/dataset/51391fb1-ae4d-44d8-9178-a06f95545604 (2019).
100 Animal Demography Unit. Coordinated Waterbird Counts (CWAC). AfrOBIS https://obis.org/dataset/a60f84ee-0e25-428e-b171-16686b554702 (2012).
101 Zettler, M. L. Macrozoobenthos baltic sea (1980-2005) as part of the IOW-Monitoring. Institut für Ostseeforschung Warnemünde, Germany https://ipt.vliz.be/eurobis/resource?r=macroben_zettler_evco (2005).
102 Milchakova, N. A., Ryabogina, V. G. & Chernyshova, E. B. Macroalgae of the Crimean coastal zone (the Black Sea, 1967-2007). Sevastopol, IBSS https://obis.org/dataset/9c30c528-b4aa-4b3c-b7c2-ff0566ff12ee (2011).
103 Addinck, W. & de Kluijver, M. North Sea observations of Crustacea, Polychaeta, Echinodermata, Mollusca and some other groups between 1986 and 2003. Expert Centre for Taxonomic Idenditification (ETI), the Netherlands https://ipt.vliz.be/eurobis/resource?r=zma_noordzee (2003).
104 Derezuyk, N. Phytoplankton of the Ukrainian Black Sea shelf (1985-2005). EurOBIS. https://ipt.vliz.be/eurobis/resource?r=derezuk_phytoplankton (accessed 2012).

105 Santos, M., Cotano, U. & Sagarminaga, Y. Zooplankton in the Bay of Biscay (1995-2004 Yearly Spring DEPM surveys). AZTI-Tecnalia. Occurrence dataset. GBIF.org https://doi.org/10.15468/1uh1hb (2020).
106 Naumov, A. Benthos of the White Sea. A database. White Sea Biological Station. Zoological Institute RAS http://ipt.vliz.be/eurobis/resource?r=white_sea_benthos (2012).
107 Markhaseva, E. L., Golikov, A. A., Agapova, T. A. & Beig, A. A. Archives of the Arctic Seas Zooplankton 1. OBIS https://obis.org/dataset/17ce69d0-2504-4ae3-bc87-16b096863f51 (1985).
108 CMAR. CSIRO Marine Data Warehouse. OBIS Australia. https://doi.org/10.15468/asbeok (accessed 2012).
109 Agència Catalana de l'Aigua. Desenvolupament d'un índex d'integritat biòtica (IBICAT) basat en l'ús dels peixos com a indicadors de la qualitat ambiental dels rius a Catalunya (Aplicació de la Directiva Marc en Política d'Aigües de la Unió Europea (2000/60/CE), 2003).
110 Agència Catalana de l'Aigua. Ajust de l'Índex d'Integritat Biòtica (IBICAT) basat en l'ús dels peixos com a indicadors de la qualitat ambiental als rius de Catalunya (Departament de Medi Ambient i Habitatge, Generalitat de Catalunya, 2010).
111 Agència Catalana de l'Aigua. ACORD GOV/139/2013, de 15 d'octubre, pel qual s'aprova el Programa de seguiment i control del Districte de conca fluvial de Catalunya per al període 2013-2018 (2018).
112 Gido, K. B., Propst, D. L., Olden, J. D. & Bestgen, K. R. Multidecadal responses of native and introduced fishes to natural and altered flow regimes in the American Southwest. *Can. J. Fish. Aquat. Sci.* **70**, 554-564 (2013).
113 Gido, K. B. et al. Pockets of resistance: Response of arid-land fish communities to climate, hydrology, and wildfire. *Freshwater Biology* **64**, 761-777 (2019).
114 Kesner, B. R. & Marsh, P. C. Central Arizona project fish monitoring: Final report. Analysis of fish population monitoring data for selected waters of the Gila River Basin, Arizona, for the five year period 2005-2009. Contract No. R09PD32013. (U.S. Bureau of Reclamation. Tempe, Arizona: Marsh and Associates, LLC., 2010).
115 Terui, A. et al. Metapopulation stability in branching river networks. *Proc. Natl. Acad. Sci. U.S.A.* **115**, E5963-E5969 (2018).
116 Iowa DNR [Department of Natural Resources]. BioNet - Iowa DNR Biological Monitoring and Assessment Database. https://programs.iowadnr.gov/bionet/ (2013).
117 Milardi, M. et al. The role of species introduction in modifying the functional diversity of native communities. *Sci. Total Environ.* **699** (2020).
118 Leveque, C., Hougard, J. M., Resh, V., Statzner, B. & Yameogo, L. Freshwater ecology and biodiversity in the tropics: what did we learn from 30 years of onchocerciasis control and the associated biomonitoring of West African rivers? *Hydrobiologia* **500**, 23-49 (2003).
119 Pyron, M., Vaughn, C. C., Winston, M. R. & Pigg, J. Fish assemblage structure from 20 years of collections in the Kiamichi River, Oklahoma. *Southwest. Nat.* **43**, 336-343 (1998).
120 Casatti, L., Ferreira, C. D. & Carvalho, F. R. Grass-dominated stream sites exhibit low fish species diversity and dominance by guppies: an assessment of two tropical pasture river basins. *Hydrobiologia* **632**, 273-283 (2009).
121 Zeni, J. O., Hoeinghaus, D. J. & Casatti, L. Effects of pasture conversion to sugarcane for biofuel production on stream fish assemblages in tropical agroecosystems. *Freshwater Biology* **62**, 2026-2038 (2017).

122 McLarney, W. O., Meador, J. & Chamblee, J. Upper Little Tennessee River biomonitoring program database. Coweeta Long Term Ecological Research Program https://coweeta.uga.edu/dbpublic/dataset_details.asp?accession54045 (2013).
123 Long Term Resource Monitoring Program. https://www.umesc.usgs.gov/data_library/fisheries/fish1_query.shtml (2016).
124 Murray-Darling Basin Authority. Murray-Darling Basin Fish and Macroinvertebrate Survey. https://data.gov.au/data/dataset/7826d7c9-bcc5-48c0-832a-66aaedfe7b0f (2018).
125 Minnesota Pollution Control Agency. Surface water data. https://cf.pca.state.mn.us/water/watershedweb/wdip/search_more.cfm?datatype=assessments (2018).
126 Montana, Fish, Wildlife & Parks, Fish Survey Sites. http://gis-mtfwp.opendata.arcgis.com/datasets/8192e75218c6460ba97ba3dd0a2fb3a5 (2019).
127 U.S. Geological Survey. BioData - Aquatic Bioassessment Data for the Nation. https://aquatic.biodata.usgs.gov/clearCriteria.action (2019).
128 U.K. Environmental Agency. National Fish Populations Database (NFPD): Freshwater fish survey relational datasets. https://data.gov.uk/dataset/d129b21c-9e59-4913-91d2-82faef1862dd/nfpd-freshwater-fish-survey-relational-datasets (2019).
129 North Carolina Department of Environmental Quality. Fish Community Assessment Data. https://deq.nc.gov/about/divisions/water-resources/water-resources-data/water-sciences-home-page/biological-assessment-branch/fish-community (2018).
130 Fagundes, D. C. et al. The stream fish fauna from three regions of the Upper Parana River basin. *Biota Neotrop* **15**, e20140187 (2015).
131 Winston, M. R., Taylor, C. M. & Pigg, J. Upstream extirpation of four minnow species due to damming of a prairie stream. *Trans. Am. Fish. Soc.* **120**, 98-105 (1991).
132 Taylor, C. M. in *Community ecology of stream fishes: concepts, approaches, and techniques* Ch. Covariation among plains stream fish assemblages, flow regimes, and patterns of water use, (American Fisheries Society Symposium, 2010).
133 Office français de la biodiversité. Suivi des éléments biologiques 'POISSONS' des rivières françaises. http://www.naiades.eaufrance.fr/acces-donnees#/hydrobiologie (2019).
134 Agencia Vasca del Agua. UBEGI. Información sobre el estado de las masas de agua de la CAPV. http://www.uragentzia.euskadi.eus/informacion/ubegi/u81-0003341/es/ (2019).
135 Davenport, S. R. Unpublished data.
136 Dala-Corte, R. B., Becker, F. G. & Melo, A. S. The importance of metacommunity processes for long-term turnover of riffle-dwelling fish assemblages depends on spatial position within a dendritic network. *Can. J. Fish. Aquat. Sci.* **74**, 101-115 (2017).
137 Toronto and Region Conservation Authority (TRCA). Watershed Fish Community. https://data.trca.ca/dataset/2018-watershed-fish-community (2018).
138 Eros, T. et al. Quantifying temporal variability in the metacommunity structure of stream fishes: the influence of non-native species and environmental drivers. *Hydrobiologia* **722**, 31-43 (2014).
139 Stefferud, J. A. Unpublished data.
140 Sers, B. Swedish Electrofishing RegiSter – SERS. Swedish University of Agricultural Sciences (SLU), Department of Aquatic Resources http://www.slu.se/elfiskeregistret (2013).

141 Benejam, L., Angermeier, P. L., Munne, A. & Garcia-Berthou, E. Assessing effects of water abstraction on fish assemblages in Mediterranean streams. *Freshwater Biology* **55**, 628-642 (2010).
142 Merciai, R., Molons-Sierra, C., Sabater, S. & Garcia-Berthou, E. Water abstraction affects abundance, size-structure and growth of two threatened cyprinid fishes. *PLoS One* **12**, e0175932 (2017).
143 Rinne, J. N. & Miller, D. Hydrology, geomorphology and management: Implications for sustainability of native southwestern fishes. *Rev. Fish. Sci.* **14**, 91-110 (2006).
144 Van Thuyne et al. VIS - Fishes in inland waters in Flanders, Belgium. Research Institute for Nature and Forest (INBO) https://www.gbif.org/dataset/823dc56e-f987-495c-98bf-43318719e30f (2013).
145 Brosens, D. et al. VIS - A database on the distribution of fishes in inland and estuarine waters in Flanders, Belgium. *Zookeys* **475**, 119-145 (2015).
146 Gammon, J. The fish communities of Big Raccoon Creek 1981-2002. Report for Heritage Environmental Services (Indianapolis, Indiana, 2013).
147 Bunn, S. E. et al. Integration of science and monitoring of river ecosystem health to guide investments in catchment protection and rehabilitation. *Freshwater Biology* **55**, 223-240 (2010).
148 Finnish electrofishing register Hertta. https://wwwp2.ymparisto.fi/koekalastus_sahko/yhteinen/Login.aspx?ReturnUrl=%2fkoekalastus_sahko (2019).
149 Whitney, J. E., Gido, K. B., Martin, E. C. & Hase, K. J. The first to arrive and the last to leave: colonisation and extinction dynamics of common and rare fishes in intermittent prairie streams. *Freshwater Biology* **61**, 1321-1334 (2016).
150 Rennie, S. et al. UK Environmental Change Network (ECN) moth data: 1992-2015. NERC Environmental Information Data Centre https://doi.org/10.5285/a2a49f47-49b3-46da-a434-bb22e524c5d2 (2017).
151 R. Van Klink et al. A global database of long-term changes in insect assemblages. Knowledge Network for Biocomplexity https://doi.org/10.5063/F1ZC817H (2020).
152 Rennie, S. et al. UK Environmental Change Network (ECN) butterfly data: 1993-2015. NERC Environmental Information Data Centre https://doi.org/10.5285/5aeda581-b4f2-4e51-b1a6-890b6b3403a3 (2017).
153 Rennie, S. et al. UK Environmental Change Network (ECN) carabid beetle data: 1992-2012. NERC Environ. Inform. Data Centre https://doi.org/10.5285/8385f864-dd41-410f-b248-028f923cb281 (2017).
154 Valtonen, A. et al. Long‐term species loss and homogenization of moth communities in Central Europe. *J. Anim. Ecol.* **86**, 730-738 (2017).
155 Pizzolotto, R., Gobbi, M. & Brandmayr, P. Changes in ground beetle assemblages above and below the treeline of the D olomites after almost 30 years (1980/2009). *Ecol. Evol.* **4**, 1284-1294 (2014).
156 Huttunen, K.-L. et al. Habitat connectivity and in-stream vegetation control temporal variability of benthic invertebrate communities. *Sci. Rep.* **7**, 1448 (2017).
157 Groker, G. National river water quality network database (macro-invertebrates). GBIF https://doi.org/10.15468/mfjetu (2018).
158 Schuch, S., Bock, J., Krause, B., Wesche, K. & Schaefer, M. Long-term population trends in three grassland insect groups: a comparative analysis of 1951 and 2009. *J. Appl. Entomol.* **135**, 321-331 (2012).
159 Schuch, S., Wesche, K. & Schaefer, M. Long-term decline in the abundance of leafhoppers and planthoppers (Auchenorrhyncha) in Central European protected dry grasslands. *Biol. Conserv.* **149**, 75-83 (2012).

160 SLU. Miljödata MVM. version 2.15.00 https://miljodata.slu.se/mvm/Default.aspx (2021).
161 Iowa Mosquito Surveillance. Iowa Mosquito Surveillance. https://mosquito.ent.iastate.edu/ (2019).
162 Marion County Health Department. Data Set: Mosquito surveillance in Marion County, Indiana, USA. VectorBase https://vectorbase.org/vectorbase/app/record/dataset/DS_9c7db23897 (2020).
163 Lee County Mosquito Control District. Data Set: Lee County Mosquito Control Department data in Lee county, Florida, 2018 VectorBase https://vectorbase.org/vectorbase/app/record/dataset/DS_8c513b1b6b (2020).
164 Manatee County Mosquito Control District. Data Set: Mosquito surveillance in Manatee County, Florida, USA. 2020 VectorBase https://vectorbase.org/vectorbase/app/record/dataset/DS_736e6c50fe (2021).
165 Pereira, F. W., Carneiro, L. & Goncalves, R. B. More losses than gains in ground-nesting bees over 60 years of urbanization. *Urban Ecosyst* **24**, 233-242 (2021).
166 Environment Agency. Ecology & Fish Bulk Downloads. UK Dep. Environ. Food Rural Aff. https://environment.data.gov.uk/ecology/explorer/downloads/ (2021).
167 Choi, S.-W. Long-term (2005–2017) macromoth community monitoring at Mt. Jirisan National Park, South Korea. https://db.cger.nies.go.jp/JaLTER/metacat/metacat?action=read&qformat=jalter-en&sessionid=&docid=ERDP-2019-02.1 (2019).
168 Choi, S. W., An, J. S., Kim, N. H., Lee, S. & Ahn, N. Long-term (2005-2017) macromoth community monitoring at Mt. Jirisan National Park, South Korea. *Ecol. Res.* **34**, 443-443 (2019).
169 Desplaines Valley Mosquito Abatement District. Data Set: Mosquito surveillance in Desplaines Valley Mosquito Abatement, Illinois, USA, 2020. VectorBase https://vectorbase.org/vectorbase/app/record/dataset/DS_ed76974f48 (2020).
170 City of Suffolk Mosquito Control. Data Set: Mosquito surveillance in City of Suffolk, Virginia, USA. 23434, 2015. VectorBase https://vectorbase.org/vectorbase/app/record/dataset/DS_a77ea39069 (2019).
171 Ada County Weed, Pest, and Mosquito Abatement. Data Set: Mosquito surveillance, Ada County Weed, Pest, and Mosquito, in Idaho, USA, 2018. VectorBase https://vectorbase.org/vectorbase/app/record/dataset/DS_da9aa55156 (2020).
172 Prendergast, J. et al. The Global Population Dynamics Database. Knowledge Network for Biocomplexity https://doi.org/10.5063/f1bz63z8 (2010).
173 Alston, J. M. et al. Ecological consequences of large herbivore exclusion in an African savanna: 12 years of data from the UHURU experiment. *Ecology* **103**, e3649 (2022).
174 Alston, J. M. et al. Ecological consequences of large herbivore exclusion in an African savanna: 12 years of data from the UHURU experiment. Dryad Dataset https://doi.org/10.5061/dryad.1g1jwstxw (2022).
175 Muthukrishnan, R. & Larkin, D. Invasive species and biotic homogenization in temperate aquatic plant communities. Dryad Dataset https://doi.org/10.5061/dryad.15dv41nt2 (2020).
176 Muthukrishnan, R. & Larkin, D. J. Invasive species and biotic homogenization in temperate aquatic plant communities. *Glob. Ecol. Biogeogr.* **29**, 656-667 (2020).
177 Myers-Smith, I. H. et al. Eighteen years of ecological monitoring reveals multiple lines of evidence for tundra vegetation change. *Ecol. Monogr.* **89**, e01351 (2019).
178 Myers-Smith, I. H. ShrubHub/QikiqtarukHub: QikiqtarukHub_v1.0. Zenodo https://doi.org/10.5281/zenodo.2397996 (2018).

179 Price, E. P. F., Spyreas, G. & Matthews, J. Biotic homogenization of regional wetland plant communities within short timescales in the presence of an aggressive invader. University of Illinois at Urbana-Champaign https://doi.org/10.13012/B2IDB-0478588_V2 (2017).
180 Santa Barbara Coastal LTER, Reed, D. & Miller., R. SBC LTER: Reef: Annual time series of biomass for kelp forest species, ongoing since 2000 ver 11. Environmental Data Initiative https://doi.org/10.6073/pasta/1a4de6e46a64186661b0407f786a14bd (2022).
181 Jeremy, R.-S. & Jacklyn, P. Three Parks Savanna Fire-effects Plot Network: Plot-based Vegetation Sampling Data, Northern Territory, Australia, 1994-2016. The Australian National University Data Commons https://doi.org/10.25911/5c3d75bbca1c0 (2017).
182 Santana, J. et al. Using beta diversity to inform agricultural policies and conservation actions on Mediterranean farmland. *J. Appl. Ecol.* **54**, 1825-1835 (2017).
183 Sonnier, G., Whittington, R. & H., B. E. Long-term response of wetland plant communities to management intensity, grazing abandonment, and prescribed fire ver 1. Environmental Data Initiative https://doi.org/10.6073/pasta/f20622c01b40e1e08e72f01e29f59302 (2022).
184 Sorte, C. J. B. et al. Long-term declines in an intertidal foundation species parallel shifts in community composition. *Glob. Change Biol.* **23**, 341-352 (2017).
185 Sperandii, M. G. & Acosta, A. T. R. Back into the past: Resurveying random plots to track community changes in Italian coastal dunes. Dryad Dataset https://doi.org/10.5061/dryad.np5hqbzr8 (2020).
186 Szydlowski, D. K., Elgin, A. K., Lodge, D. M., Tiemann, J. S. & Larson., E. R. Aquatic snail and macrophyte abundance and richness data for ten lakes in Vilas County, WI, USA, 1987-2020 ver 1. Environmental Data Initiative https://doi.org/10.6073/pasta/29733b5269efe990c3d2d916453fe4dd (2022).
187 Tanner, J. E. & Connell, J. H. Coral community data Heron Island Great Barrier Reef 1962–2016. *Sci Data* **9**, 617 (2022).
188 Vojik, M. & Boublik, K. Fear of the dark: decline in plant diversity and invasion of alien species due to increased tree canopy density and eutrophication in lowland woodlands. *Plant Ecol.* **219**, 749-758 (2018).
189 Wardle, G., Dickman, C., Greenville, A. & Tamayo, B. Desert Ecology Plot Network: Vegetation Plot-data, Simpson Desert, Western Queensland, 1993-2018. Long Term Ecological Research Network (LTERN), ANU Data Commons, The Australian National University https://doi.org/10.25911/5c355ce264ffe (2018).
190 Wardle, G., Dickman, C., Greenville, A. & Tamayo, B. Desert Ecology Plot Network: Reptile Abundance Plot-data, Simpson Desert, Western Queensland, 1990-2018. Long Term Ecological Research Network (LTERN), ANU Data Commons, The Australian National University http://doi.org/10.25911/5c1099272c24b (2018).
191 Willig, M. R. El Verde long-term invertebrate data - Luquillo Forest Dynamics Plot (LFDP) ver 9996737. Environmental Data Initiative https://doi.org/10.6073/pasta/45e3a90ed462f66acdde83636746f87f (2020).
192 Wright, K. W. et al. Bee species abundance and composition in three ecosystem types at the Sevilleta National Wildlife Refuge, New Mexico, USA ver 2. Environmental Data Initiative https://doi.org/10.6073/pasta/cbe04a94b5f6f3859a3d9c98f5be0fc8 (2021).
193 Alves, C. et al. Twenty years of change in benthic communities across the Belizean Barrier Reef. *PLoS One* **17**, e0249155 (2022).

194 Amesbury, S. S., Ginsburg, D., Rongo, T., Kirkendale, L. & Starmer, J. War-in-the-pacific national historical park marine biological survey, a Report to the U.S. National Park Service. University of Guam Marine Laboratory (1999).

195 Gruman, C. et al. Replication data for: Long term monitoring of the rocky intertidal on Wizard Island in Barkley Sound. Scholars Portal Dataverse, V1.1 https://doi.org/10.5683/SP2/VBPBFN (2019).

196 Beldade, R., Mills, S. C., Claudet, J. & Cote, I. M. More coral, more fish? Contrasting snapshots froma remote Pacific atoll. *PeerJ* **3**, e745 (2015).

197 Burlakova, L. E. et al. Density data for Lake Ontario benthic invertebrate assemblages from 1964 to 2018. *Ecology* **102**, e03528 (2021).

198 Cervantes-Yoshida, K., Leidy, R. A. & Carlson, S. M. Contemporary Land Change Alters Fish Communities in a San Francisco Bay Watershed, California, USA. *PLoS One* **10**, e0141707 (2015).

199 Chen, I. C. et al. Asymmetric boundary shifts of tropical montane Lepidoptera over four decades of climate warming. *Glob. Ecol. Biogeogr.* **20**, 34-45 (2011).

200 Christensen, E. et al. Quadrat-based monitoring of desert grassland vegetation at the Jornada Experimental Range, New Mexico, 1915-2016. *Ecology* **102**, e03530 (2021).

201 Closset-Kopp, D., Hattab, T. & Decocq, G. Do drivers of forestry vehicles also drive herb layer changes (1970-2015) in a temperate forest with contrasting habitat and management conditions? *J. Ecol.* **107**, 1439-1456 (2019).

202 Dunbar, M., Murphy, J., Clarke, R., Davies, C. & Scarlett, P. Headwater stream invertebrate data 2007 [Countryside Survey]. NERC Environmental Information Data Centre https://doi.org/10.5285/18849325-358b-4af1-b20d-d750b1c723a3 (2016).

203 Furse, M. T. et al. Headwater stream invertebrate data 1990 [Countryside Survey]. NERC Environmental Information Data Centre https://doi.org/10.5285/b4c17f35-1b50-4ed7-87d2-b63004a96ca2 (2016).

204 Furse, M. T. et al. Headwater stream invertebrate data 1998 [Countryside Survey]. NERC Environmental Information Data Centre https://doi.org/10.5285/fd0ce233-3b4d-4a5e-abcb-c0a26dd71c95 (2016).

205 Dunbar, M., Murphy, J., Clarke, R., Davies, C. & Scarlett, P. Headwater stream macrophyte data 2007 [Countryside Survey]. NERC Environmental Information Data Centre https://doi.org/10.5285/249a90ec-238b-4038-a706-6633c3690d20 (2016).

206 Furse, M. T. et al. Headwater stream macrophyte data 1998 [Countryside Survey]. NERC Environmental Information Data Centre https://doi.org/10.5285/e0b638d5-8271-4442-97ef-cf46ea220f5d (2016).

207 Wood, C. M. et al. Long-term vegetation monitoring in Great Britain - the Countryside Survey 1978-2007 and beyond. *Earth Syst Sci Data* **9**, 445-459 (2017).

208 Santa Barbara Coastal LTER & Dugan, J. SBC LTER: Beach: Time series of abundance of birds and stranded kelp on selected beaches, ongoing since 2008 ver 10. Environmental Data Initiative https://doi.org/10.6073/pasta/06c6b9983a5f0a44349e027a002f5040 (2021).

209 Ellingsen, K. E., Yoccoz, N. G., Tveraa, T., Hewitt, J. E. & Thrush, S. F. Data from: Long-term environmental monitoring for assessment of change: measurement inconsistencies over time and potential solutions. Dryad https://doi.org/10.5061/dryad.2v7m4 (2018).

210 Gaget, E. et al. Disentangling the latitudinal and altitudinal shifts in community composition induced by climate change: the case of riparian birds. Dryad Dataset https://doi.org/10.5061/dryad.1rn8pk0rx (2021).

211 Gibb, H., Grossman, B., Dickman, C., Decker, O. & Wardle, G. Data from: Long-term responses of desert ant assemblages to climate. Dryad Dataset https://doi.org/10.5061/dryad.vc80r13 (2019).
212 Green, J. M., Dunbrack, R. L. & Bates, A. E. Signals of resilience and change in tidepool fish communities on the Pacific coast of Vancouver Island, Canada. *Divers. Distrib.* **27**, 2170-2179 (2021).
213 Keith, D. et al. Upland Heath Swamps Plot Network: Vegetation Floristics, Royal National Park, Sydney Basin, NSW, Australia, 1990-2014. Long Term Ecological Research Network https://doi.org/10.25911/5c36e061dc7da (2018).
214 Kolesnikova, A., Konakova, T., Taskaeva, A. & Kudrin, A. Soil invertebrates of coniferous forests along a gradient of air pollution (Komi Republic). *Biodiversity Data Journal* **9** (2021).
215 Larkin, D. J. et al. Phylogenetic measures of plant communities show long-term change and impacts of fire management in tallgrass prairie remnants. *J. Appl. Ecol.* **52**, 1638-1648 (2015).
216 Lelli, C. et al. Long-term changes in Italian mountain forests detected by resurvey of historical vegetation data. *J Veg Sci* **32**, e12939 (2021).
217 Lightfoot, D. & Whitford., W. Lizard pitfall trap data from 11 NPP study locations at the Jornada Basin LTER site, 1989-2006. Environmental Data Initiative https://doi.org/10.6073/pasta/51814fa39f87aea44629a5be8602ec49 (2022).
218 Magalhaes, A. L. B. et al. All the colors of the world: biotic homogenization-differentiation dynamics of freshwater fish communities on demand of the Brazilian aquarium trade. *Hydrobiologia* **847**, 3897-3915 (2020).
219 Maleki, K., Marchand, P., Charron, D., Lafleur, B. & Bergeron, Y. A 249‐yr chronosequence of forest plots from eight successive fires in the Eastern Canada boreal mixedwoods. *Ecology* **102**, e03306 (2021).
220 Matthews, W. J. & Marsh-Matthews, E. Dynamics of an upland stream fish community over 40 years: trajectories and support for the loose equilibrium concept. *Ecology* **97**, 706-719 (2016).
221 Mendieta‐Leiva, G., Buckley, H. L. & Zotz, G. Directional changes over time in the species composition of tropical vascular epiphyte assemblages. *J. Ecol.* **110**, 553-568 (2022).
222 Moore, M. M. et al. Cover and density of southwestern ponderosa pine understory plants in permanent chart quadrats (2002–2020). *Ecology* **103**, e3661 (2022).
223 Mushet, D. M., Euliss, N. H., Jr. & Solensky, M. J. Cottonwood Lake Study Area - Invertebrate Counts. U.S. Geological Survey data release https://doi.org/10.5066/F7BK1B77 (2017).
