# Supplementary material for: Regional occupancy increases for widespread species but decreases for narrowly distributed species in metacommunity time series
Source: Nat Commun. 2023 Mar 16;14:1463. doi: 10.1038/s41467-023-37127-2 (PMC10020147; doi:10.1038/s41467-023-37127-2)
Supplement: Supplementary file 3 — Reporting Summary [file 41467_2023_37127_MOESM3_ESM.pdf]

nature portfolio

Corresponding author(s): Wu-Bing Xu, Jonathan M. Chase

Last updated by author(s): Feb 24, 2023



# Reporting Summary

Nature Portfolio wishes to improve the reproducibility of the work that we publish. This form provides structure for consistency and transparency in reporting. For further information on Nature Portfolio policies, see our Editorial Policies and the Editorial Policy Checklist.

## Statistics

For all statistical analyses, confirm that the following items are present in the figure legend, table legend, main text, or Methods section.

| n/a | Confirmed | |
|---|---|---|
| ☐ | ☒ | The exact sample size (*n*) for each experimental group/condition, given as a discrete number and unit of measurement |
| ☐ | ☒ | A statement on whether measurements were taken from distinct samples or whether the same sample was measured repeatedly |
| ☐ | ☒ | The statistical test(s) used AND whether they are one- or two-sided<br>*Only common tests should be described solely by name; describe more complex techniques in the Methods section.* |
| ☐ | ☒ | A description of all covariates tested |
| ☐ | ☒ | A description of any assumptions or corrections, such as tests of normality and adjustment for multiple comparisons |
| ☐ | ☒ | A full description of the statistical parameters including central tendency (e.g. means) or other basic estimates (e.g. regression coefficient) AND variation (e.g. standard deviation) or associated estimates of uncertainty (e.g. confidence intervals) |
| ☐ | ☒ | For null hypothesis testing, the test statistic (e.g. *F*, *t*, *r*) with confidence intervals, effect sizes, degrees of freedom and *P* value noted<br>*Give P values as exact values whenever suitable.* |
| ☐ | ☒ | For Bayesian analysis, information on the choice of priors and Markov chain Monte Carlo settings |
| ☐ | ☒ | For hierarchical and complex designs, identification of the appropriate level for tests and full reporting of outcomes |
| ☐ | ☒ | Estimates of effect sizes (e.g. Cohen's *d*, Pearson's *r*), indicating how they were calculated |

*Our web collection on statistics for biologists contains articles on many of the points above.*

## Software and code

Policy information about availability of computer code

| | |
|---|---|
| Data collection | We used a number of computational resources in R to compile, clean and organize data from open-access databases. The package “rgbif (v. 3.6.0)” was used to compile species distribution occurrences from the Global Biodiversity Information Facility; the package “CoordinateCleaner (v. 2.0-18)” was used to clean distribution occurrences. All code used in this study has been deposited at GitHub (https://github.com/Wubing-Xu/Range_size_winners_losers) and mirrored at Zendo (https://doi.org/10.5281/zenodo.7675355). |
| Data analysis | We used a number of computational resources in R to do data analysis. The R package “alphahull (v. 2.2)” was used to construct alpha hulls; the R package “brms (v. 2.15.0)” was used to fit Bayesian models. All code for analyses has been deposited at GitHub (https://github.com/Wubing-Xu/Range_size_winners_losers) and mirrored at Zendo (https://doi.org/10.5281/zenodo.7675355). |

For manuscripts utilizing custom algorithms or software that are central to the research but not yet described in published literature, software must be made available to editors and reviewers. We strongly encourage code deposition in a community repository (e.g. GitHub). See the Nature Portfolio guidelines for submitting code & software for further information.

## Data

Policy information about availability of data

All manuscripts must include a data availability statement. This statement should provide the following information, where applicable:

- Accession codes, unique identifiers, or web links for publicly available datasets
- A description of any restrictions on data availability
- For clinical datasets or third party data, please ensure that the statement adheres to our policy

All of the data used in this analysis are open-access and available on GitHub (https://github.com/Wubing-Xu/Range_size_winners_losers), and are mirrored on Zenodo (https://doi.org/10.5281/zenodo.7675355). Additionally, the original sources of data used in this study are publicly available. The BioTIME data can be accessed on Zenodo (https://doi.org/10.5281/zenodo.2602708) or through the BioTIME website (https://biotime.st-andrews.ac.uk/); the RivFishTIME data can be accessed through the iDiv Biodiversity Portal (https://doi.org/10.25829/idiv.1873-10-4000); the InsectChange data can be accessed on KNB (https://doi.org/10.5063/F11V5C9V) or through the data paper (http://onlinelibrary.wiley.com/doi/10.1002/ecy.3354/suppinfo); the 'Metacommunity Resurveys' data can be accessed through the iDiv Biodiversity Portal (https://doi.org/10.25829/idiv.3503-jevu6s); the species occurrences are available on Global Biodiversity Information Facility (https://doi.org/10.15468/dl.6vdkb); the protected area data are available on World Database on Protected Areas (accessed October 2022; https://www.protectedplanet.net/en/thematic-areas/wdpa?tab=WDP).

## Human research participants

Policy information about studies involving human research participants and Sex and Gender in Research.

| | |
|---|---|
| Reporting on sex and gender | N/A |
| Population characteristics | N/A |
| Recruitment | N/A |
| Ethics oversight | N/A |

Note that full information on the approval of the study protocol must also be provided in the manuscript.

# Field-specific reporting

Please select the one below that is the best fit for your research. If you are not sure, read the appropriate sections before making your selection.

☐ Life sciences ☐ Behavioural & social sciences ☒ Ecological, evolutionary & environmental sciences

For a reference copy of the document with all sections, see nature.com/documents/nr-reporting-summary-flat.pdf

# Ecological, evolutionary & environmental sciences study design

All studies must disclose on these points even when the disclosure is negative.

| | |
|---|---|
| Study description | This study compiled 238 datasets of metacommunity time series examining the relationship between species' geographic range size and changes in species occupancy (proportion of sites where a species is present) within a metacommunity through time. Further, this study also examined whether the effect of range size on occupancy changes is reduced in regions that are largely protected compared to non-protected regions. |
| Research sample | Because this study focused on temporal changes of species occupancy within a metacommunity (i.e., multiple localities within a region), we used metacommunity time series that had at least four local sites sampled at each year, spanning at least 10 years between the first and last sampling date. We further selected metacommunity time series that has at least 10 species because this study aimed to test the relationship between species' range size and occupancy change within metacommunities. There were 81 terrestrial, 68 freshwater and 88 marine metacommunity time series. The data used in this study came from four open-access databases: BioTIME, RivFishTIME, InsectChange, and Metacommunity Resurveys. See the "Data" section in this Reporting Summary and the "Methods" section in the paper for details on the data. |
| Sampling strategy | Because this was a quantitative synthesis, the raw studies differed widely in their sampling methodology. But for each study, data of assemblages were sampled using a similar methodology through time. As the number and spatial configuration of sites in each dataset can be different across years, we developed an approach to match sites through time. In this study, a total of 238 datasets were compiled, which is sufficient for a quantitative synthesis. |
| Data collection | As this was a quantitative synthesis of 238 studies of multiple groups of organisms, many hundreds of people were involved in the data collection. The specific investigators and investigating procedures can be found in original studies, which were presented in the publications and repositories of four used databases: BioTIME, RivFishTIME, InsectChange, and Metacommunity Resurveys. |
| Timing and spatial scale | As a quantitative synthesis, the time period across collected datasets ranged from 1927 until 2021, with a median start year of 1994. |

| | |
|---|---|
| | The duration of data collection ranged from 10 to 90 years, with a median of 16 years. The number of local sites ranged from 4 to 6308 sites at each time point, with a median of 26 sites. The spatial extent ranged from 0.001 to 22,695,462 km2, with a median of 4,274 km2. |
| Data exclusions | No Data were excluded from the analyses. |
| Reproducibility | This study was based on analyses of 238 metacommunity time series, which came from open-access databases as described above. No experiments were performed for this study. All methods on data integration, processing and analyses were described in detail in the manuscript. All datasets and R codes used to produce the results in this study have been deposited on GitHub (https://github.com/Wubing-Xu/Range_size_winners_losers) and mirrored at Zendo (https://doi.org/10.5281/zenodo.7675355). |
| Randomization | The data used in this study was metacommunity time series. Across time points, the number and spatial configuration of sites in raw studies can be different because of differential sampling efforts through time in some studies. To make samples comparable across years, we developed an approach to match sites through time. We chose sites in similar spatial configurations across years and used sample-based rarefaction to account for the variation in sampling efforts. |
| Blinding | Blinding was relevant to this study because investigators collected assemblages using a similar methodology through the years. |

Did the study involve field work? ☐ Yes ☒ No

# Reporting for specific materials, systems and methods

We require information from authors about some types of materials, experimental systems and methods used in many studies. Here, indicate whether each material, system or method listed is relevant to your study. If you are not sure if a list item applies to your research, read the appropriate section before selecting a response.

## Materials & experimental systems

| n/a | Involved in the study |
|---|---|
| ☒ | ☐ Antibodies |
| ☒ | ☐ Eukaryotic cell lines |
| ☒ | ☐ Palaeontology and archaeology |
| ☒ | ☐ Animals and other organisms |
| ☒ | ☐ Clinical data |
| ☒ | ☐ Dual use research of concern |

## Methods

| n/a | Involved in the study |
|---|---|
| ☒ | ☐ ChIP-seq |
| ☒ | ☐ Flow cytometry |
| ☒ | ☐ MRI-based neuroimaging |
